# Physical Motif Clustering within Intrinsically Disordered Nucleoporin Sequences Reveals Universal Functional Features

**DOI:** 10.1371/journal.pone.0073831

**Published:** 2013-09-16

**Authors:** David Ando, Michael Colvin, Michael Rexach, Ajay Gopinathan

**Affiliations:** 1 Physics Department, University of California Merced, Merced, California, United States of America; 2 Chemistry and Chemical Biology, University of California Merced, Merced, California, United States of America; 3 Molecular, Cell, and Developmental Biology, University of California Santa Cruz, Santa Cruz, California, United States of America; Weizmann Institute of Science, Israel

## Abstract

Bioinformatics of disordered proteins is especially challenging given high mutation rates for homologous proteins and that functionality may not be strongly related to sequence. Here we have performed a novel bioinformatic analysis, based on the spatial clustering of physically relevant features such as binding motifs and charges within disordered proteins, on thousands of Nuclear Pore Complex (NPC) FG motif containing proteins (FG nups). The biophysical mechanism by which FG nups regulate nucleocytoplasmic transport has remained elusive. Our analysis revealed a set of highly conserved spatial features in the sequence structure of individual FG nups, such as the separation, localization, and ordering of FG motifs and charged residues along the protein chain. These functionally conserved features provide insight into the particular biophysical mechanisms responsible for regulation of nucleocytoplasmic traffic in the NPC, strongly constraining current models. Additionally this method allows us to identify potentially functionally analogous disordered proteins across distantly related species.

## Introduction

Nuclear pore complexes are large supramolecular structures 40 to 120 nm in size composed of numerous copies of over 30 nucleoporin (nup) proteins. Each complex spans the nuclear envelope and conducts highly selective regulation of transport between the nucleus and cytoplasm, including critical cargos such as mRNA and gene regulatory proteins [1]–[3].

Although small molecules are free to diffuse through the NPC, cargoes larger than around 40 kDa are required to bind with karyopherins receptor proteins (kaps) to cross the NPC permeability barrier [4], [5]. Kaps facilitate transport via binding to numerous FG motifs [6], [7] within largely disordered FG nup proteins distributed around and throughout the central channel of the NPC. These FG nups accomplish selective gating by forming a permeability barrier whose organization, structure, and dynamics are still unknown and highly debated.

Currently, it is known that the central channel of the NPC is surrounded by a cylindrical scaffold consisting of tightly-interwoven structural proteins (around two thirds of NPC proteins) and the folded domains of FG nups, which serve as anchors to this scaffold [3].

Bioinformatic and proteomic analyses of the NPC have been successful in determining the structure, function, and origin of these structural elements of the pore [3], [8]. The disordered regions of nups have on the other hand generated much debate with regards to their underlying biophysical properties and function. Experimental results on FG nup aggregation and function are not all consistent [9]–[11], and traditional bioinformatics tools are unsuitable to predict function for these disordered proteins primarily due to their high AA substitution rates [12].

The reason for this is that typical bioinformatic sequence analysis involves pairwise comparison of amino acids (AAs) to reveal relationships among separate proteins [13], but this implicitly relies on a strong correlation between sequence, structure and function. However, since individual disordered proteins have the ability to form large functional ensembles of structures rather than a single folded structural state, they are much more robust to substantial changes in sequence over and above BLOSUM [14] or PAM [15] type similarity mutations, with often very-dissimilar sequences producing identical function [16]. In essence, it is not clear how disordered proteins retain functionality in the absence of a specific structure or a conserved sequence, both of which are normally associated with function in proteins [17].

It is therefore possible that the spatial distribution of physical properties along the disordered protein chain is a more relevant determinant of function than the specific AA sequence. Such an approach has been successful with other disordered protein systems such as the comprehensive analysis of 1,384 wild-type homeodomains by Vuzman *et al* [18]. They showed that the charge composition and distribution are evolutionary conserved, with positively charged amino acids forming dense and large clusters in order to functionally enhance binding to negatively charged DNA. This is a clear example that the sequence to structure to function paradigm well known among structured proteins can be applied at a coarse-grained level to intrinsically disordered proteins as well. Additionally, the general relationship between the sequence composition of disordered proteins, their structural characteristics, and function is currently a major area of research [19]. For example, Vucetic *et al* [20] have been able to broadly classify disordered protein sequences using a combination of sequence analysis tools into three groups arbitrarily labeled Flavor V, C, and S. Flavor V sequences tend to have positive charge and function as ribosomal proteins, Flavor C proteins tend to be neutral and function as modification sites, while Flavor S proteins tend to be negatively charged and function in protein binding.

Here we have taken the approach of analyzing the distribution of AA physico-chemical properties along FG nup polymers to model the copolymer block structure of these properties in order to generate metrics for comparison between FG nups that overcome highly divergent and noisy sequence data to reveal functional features. This approach also allows us to use these features to separate FG nups into distinct groups with potentially distinct functions, and to identify analogous subsets of these proteins among different species.

We assembled a data set for analysis of 3,355 nuclear pore related sequences (Data S1) tagged with the keyword “nucleoporin” from the Universal Protein Database [21]. This data set contained FG nups from 252 species (Text S1). These proteins were characterized by the percentage of probable disorder as predicted by the PONDR algorithm [22] and by their FG motif distribution density (FG motifs/AA). Two groups arose naturally (Text S1, Figure S2, Figure S3, Figure S4, Figure S5); one with low percentage disorder and FG density, which we identified as structural nups and kaps, and another group with relatively high percentage disorder and FG density, the FG nups. We focus on the second group for the rest of this analysis.

**Figure 1:**
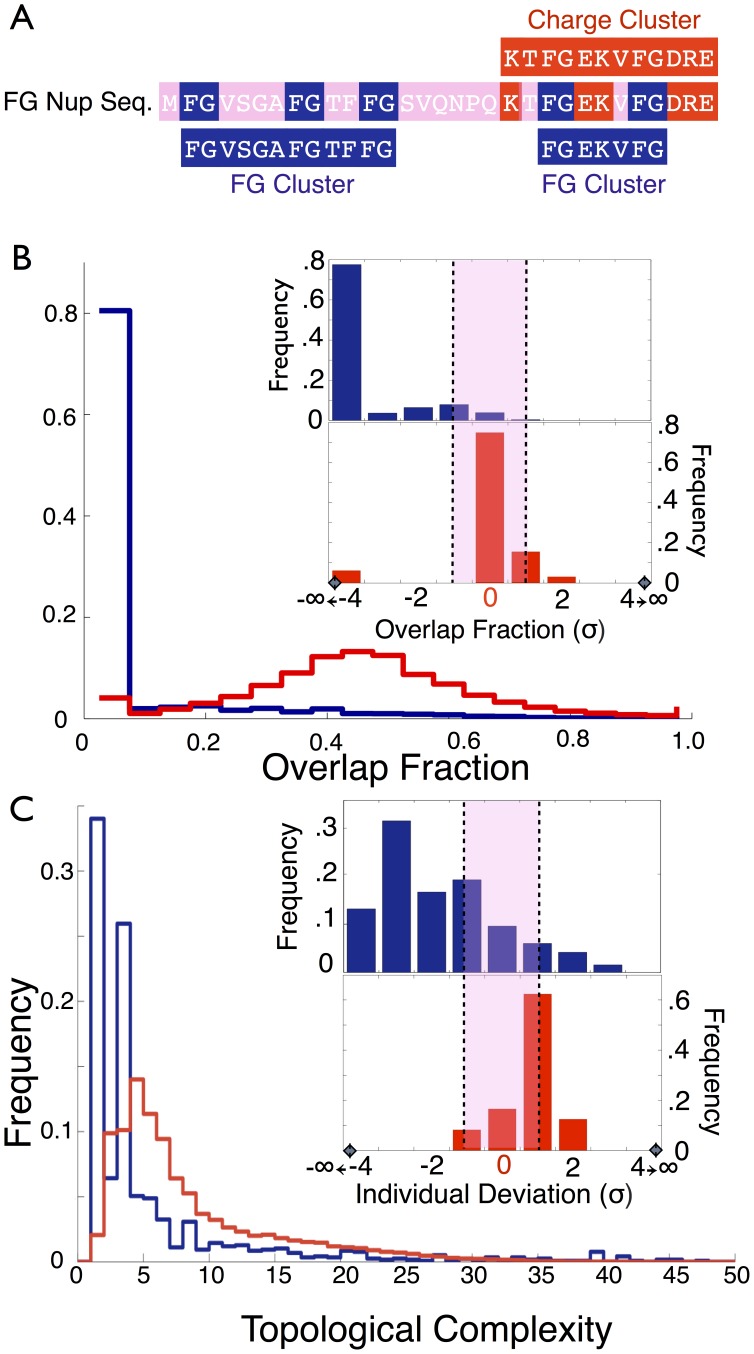
Spatial relationship between FG and charge clusters. (A) The FG clusters and charge clusters of an example FG nup sequence. (B) Percent overlap of FG clusters with charge clusters (blue). Almost 80 percent of FG motif cluster regions have zero overlap with clusters of charges. Other percentages of overlap, while strongly in the minority, appear with roughly equal probability. FG nups which have been randomly shuffled (in red) have 4 percent of their FG motif cluster regions completely disjoint from charge clusters while there is a strong tendency for FG clusters to overlap with charge clusters with a most probable frequency overlap at 45 percent. Inset shows statistical significance of degree of overlap for FG nups (top, blue) versus WG/FG control group (bottom, red). Horizontal axis in inset shows the deviation of the percent overlap from the mean value for the randomly shuffled ensemble measured in units of the standard deviation of the random ensemble distribution. (B) Histogram of the topological complexity of FG nups (in blue) and randomly shuffled nups (in red). A majority of FG nups (66%) have a low topological complexity (less than 4) with 34% being purely diblock charge-FG copolymer structure, while randomly shuffled nups have only a small minority (22%) with low topological complexity and only 2% are diblocks. Upper inset shows that 77% of FG nups have a topological complexity which is less than their random ensemble by more than one standard deviation, while the control group shows little deviation from the ensemble (red, lower inset).

In this paper we use an approach called “density-based clustering” where density is the number of occurrences of a specific motif (e.g. “FG” AA pair or charged AA) per residue in a given segment of the protein. A cluster of a particular motif is defined as a segment of the protein with a higher density of that motif than the remainder of its amino acid sequence which models the copolymer block distribution for that motif, as depicted in Figure 1A. Note that individual occurrences of motifs appearing in low-density regions that separate clusters are considered to be noise in this clustering scheme. For this paper we adopted the PreDeCon density-clustering algorithm because it is determinate, robust against noise, efficient, and shows a superior clustering accuracy over other relevant methods [23]. We used a form of unsupervised clustering (see Methods) which determines the optimal clustering [24], via the Dunn index validity measure [25].

We first clustered two easily-defined, physico-chemical motifs within individual FG nups, the FG binding motifs and charged AAs, using our density clustering algorithm. We chose these two features because they are clearly defined and have been previously associated with FG nup shape and function [26], [27]. The FG sequence motif has been extensively studied and strongly implicated in transport factor binding [6], [7], [28], whereas the density of charged amino acids has been frequently noted as a predictor of intrinsically-disordered proteins and is generally an important predictor of polymer properties. For the sake of completeness, we also explored clustering across a wide spectrum of possible alternative motif choices (Text S1, Figure S1), and concluded that the distribution of FG motifs and charged AA residues yielded the easiest to interpret results.

## Results

### Motif Clustering

We applied our clustering algorithm to the entire set of FG nups in all species studied to identify clusters that are enhanced in FG motifs and charged amino acids. FG clusters contained on average around 103 AAs, while charge clusters were smaller containing around 25 AAs on average. In analyzing these clusters we found that in all cases the FG nups have roughly 80% of their FG motif clusters located in regions which have absolutely no overlap with regions where charged AA clusters are found. This finding is shown in Figure 1B, which plots a histogram of the percent overlap in the FG and charged AA clusters (shown in blue). In order to examine the significance of this result for individual proteins, we generated a control ensemble of proteins consisting of random shufflings of each sequence (see Methods) and calculated the overlap of the FG motif and charged AA clusters in this ensemble; the results are plotted in Figure 1B (shown in red). For each of the FG nups we computed the number of standard deviations, 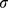 that the overlap value is away from the mean overlap value of the shuffled ensemble (Figure 1B, inset, shown in blue). Approximately 90% of FG nups had an overlap that was one or more standard deviations less than their ensemble mean, and 77% had an overlap more than three standard deviations less than the ensemble average. In contrast the vast majority of FG clusters in the randomly-shuffled ensemble had large degrees of overlap with charge clusters, with only 4% having no overlap.

We also created a control group of 24 FG-nup-like proteins (Data S1), composed of WG motif argonaute binding proteins and non-nucleoporin FG repeat proteins, which share numerous similarities to FG nups including substantial amounts of disorder and a binding motif. Argonaute binding proteins contain WG motifs within their disordered domains, which are functional in substrate binding [29] similar to how FG motifs bind to karyopherins. An additional twelve non-nucleoporin FG-motif containing proteins involved in protein transport and sorting (similar to FG nups) were obtained from an exhaustive search of the *Saccharomyces cerevisiae* genome [30]. Notably, the control group exhibited little difference from the random ensemble in terms of FG (or WG) cluster overlap with the charged AA clusters, with greater than 85% of proteins having an overlap that was one standard deviation or less from the mean of the random ensemble (Figure 1B, inset, shown in red).

Although FG and charge clusters are highly disjoint, the ordering of these clusters along the protein chain can display various levels of spatial organization. To quantify this higher level cluster organization, we defined the topological complexity as the number of times that FG and charge clusters alternate with each other along the entire length of the amino acid polymer (see Methods for details). For example, a diblock polymer (i.e. a protein with a single cluster of charged AAs next to a single cluster of FG motifs) would alternate once between cluster types along the polymer resulting in a topological complexity of 1.

We found that over one third of FG nups adopt a simple diblock topological complexity of 1 (see Figure 1C, blue histogram), and another quarter of them adopt a topological complexity of 3, a quad-block structure (ABAB). In general, the distribution of the topological complexities of the FG nups is highly skewed, with 60% of FG nups having a topological complexity which is less than the overall mean by one or more standard deviations and 11% having a topological complexity greater than one standard deviation above their ensemble. In stark contrast to the FG nups is the control group which stands essentially indistinguishable from the random ensemble in terms of topological complexity, with more than 75% of proteins differing from the random ensemble by less than one standard deviation. The ordering of FG clusters relative to charged AA clusters is therefore very different than what would result from chance alone, suggesting a functional role.

### Relative Orientations

Given our result that the vast majority of FG nups have well separated charged AA and FG motif clusters, we explored the distribution of these clusters relative to the N- and C-termini. We defined the FG-charge polarity, 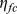, as the ratio of the distance between the centers of mass of FG and charged AA clusters to the total nup length, normalized such that 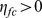 indicates a charge to FG orientation coincident with the N-to-C terminus direction. This value ranges from 0 if for example the FG and charged AA motif clusters are wholly overlapping or interspaced, to values of 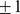 if the regions are disjoint and at opposite ends of the protein. Positive values of 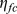 are therefore indicative of situations where the FG motifs cluster towards the C-terminus of the protein.

In the sequences analyzed, we found that FG nups have a strong tendency for 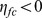, with 70% having 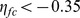. This indicates that FG clusters are preferentially located more towards the N-terminus of the protein than the charged AA clusters, and the centers of mass of the two motif clusters are separated by about half the protein length. Comparing to the results for the randomly-shuffled ensembles, we find 77% of FG nups having a lower (more negative) FG-charge polarity than the random ensemble mean by more than one standard deviation. In contrast, the FG-charge polarity computed for the control group of proteins resembles the random ensemble. Polarity for the randomly-shuffled ensembles of the FG nups showed no overall average polarity, as expected by symmetry.

**Figure 2:**
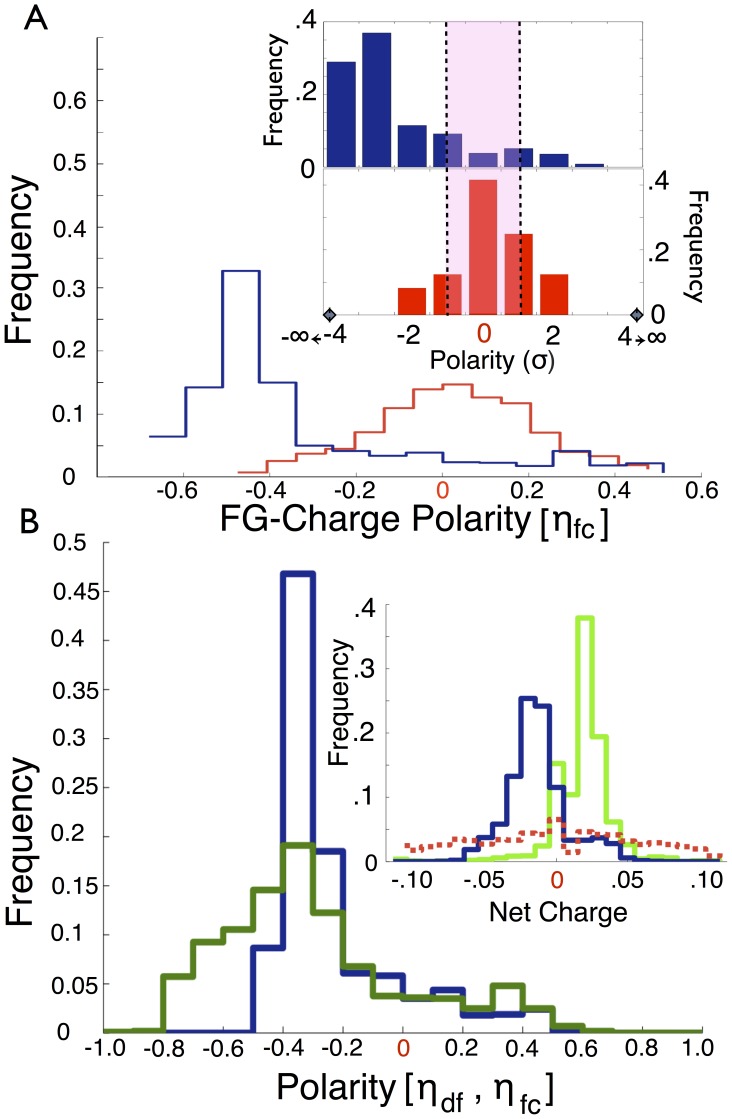
Polarity and charges of FG nups. (A) Polarity 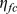 between charge and FG regions. FG nups (blue) tend to adopt a large negative and well conserved value for N-terminus to C-terminus polarity. Randomly shuffled FG nups showed no overall average polarity (red) and the statistical significance of FG nup polarity was consistently higher than three standard deviations (inset, blue, upper). The control group did not show a considerable difference from the random ensemble (inset, red, lower). (B) Polarity 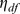 between disordered and folded regions (blue) using the PONDR [22] protein disorder predictor. Observed polarities are on average similar to FG to charge cluster polarities, 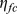. Interestingly, 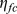 values for the disordered regions alone (green) show a similar trend. Inset shows the net charge of folded structural nups/kaps (blue) and FG clusters (green) which appear to be equal and opposite on the whole, while disordered charge cluster regions (red dashed) appear to be net neutral. Histogram values for net charges for charge clusters greater than 0.1 e/AA and less than −0.1 e/AA are negligible and are shown in Text S1.

The high conservation of the net negative FG-charge polarity (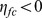) across a wide range of species suggests that this property is conserved by evolution and presumably related to NPC function. To relate this polarity to the spatial orientation of the FG nups in the NPC, we similarly looked at the polarity, 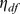, between disordered and folded regions considering that the folded regions are predominantly anchor domains that connect the nups to the NPC scaffold [26]. The observed polarities were (Figure 2B) very similar to the average FG-charge cluster polarities, indicating that the clustering of the FG motifs is nearly always towards the N-terminal tip of the nups in disordered regions, away from an anchor domain which attaches to the NPC scaffold inner wall. Interestingly, FG-charge polarity is also found to be maintained locally within disordered regions for FG nups that contain at least one charge cluster in a disordered region, indicating a charge-containing spacer sequence (or entropic chain) closer to the wall, while a minority of FG nups had no charge clusters in their disordered regions (Figure 2B). This is consistent with the bimodal distribution of these features in nups from *S. cerevisiae* [26].

It is interesting to note that since mRNA translation into protein is initiated at the N-terminus, the observed disorder-folded polarity implies that the vast majority of nups have the disordered region synthesized prior to the ordered portion. This may have implications for the processing and transport of newly synthesized nups.

### Net Charges of FG Nup Regions

We next analyzed the exact distribution of charged AAs in FG nups and found that the net charge of FG clusters (Figure 2B, inset) is positive, and nearly equal and opposite to the net charge of kaps and structural nups. We also find that the disordered regions of FG nups, which exclude FG clusters but contain charge clusters (Text S1, Figure S6), are neutral on average. The folded regions of FG nups on their own were also found to have zero net charge on average, but to associate with structural nups which are negatively-charged (Text S1, Figure S7).

Previous results regarding net charges of nucleoporins by Ribbeck *et al* are consistent but less specific, showing that for two species, *S. cerevisiae* and humans, kaps are strongly biased towards having negative total charge [31], while the disordered regions of FG nups have a tendency to be positively-charged. We show this trend is common throughout all eukaryotic species, and more significantly that the positive charges are localized to the FG domains of N-terminal tips of disordered regions.

**Figure 3:**
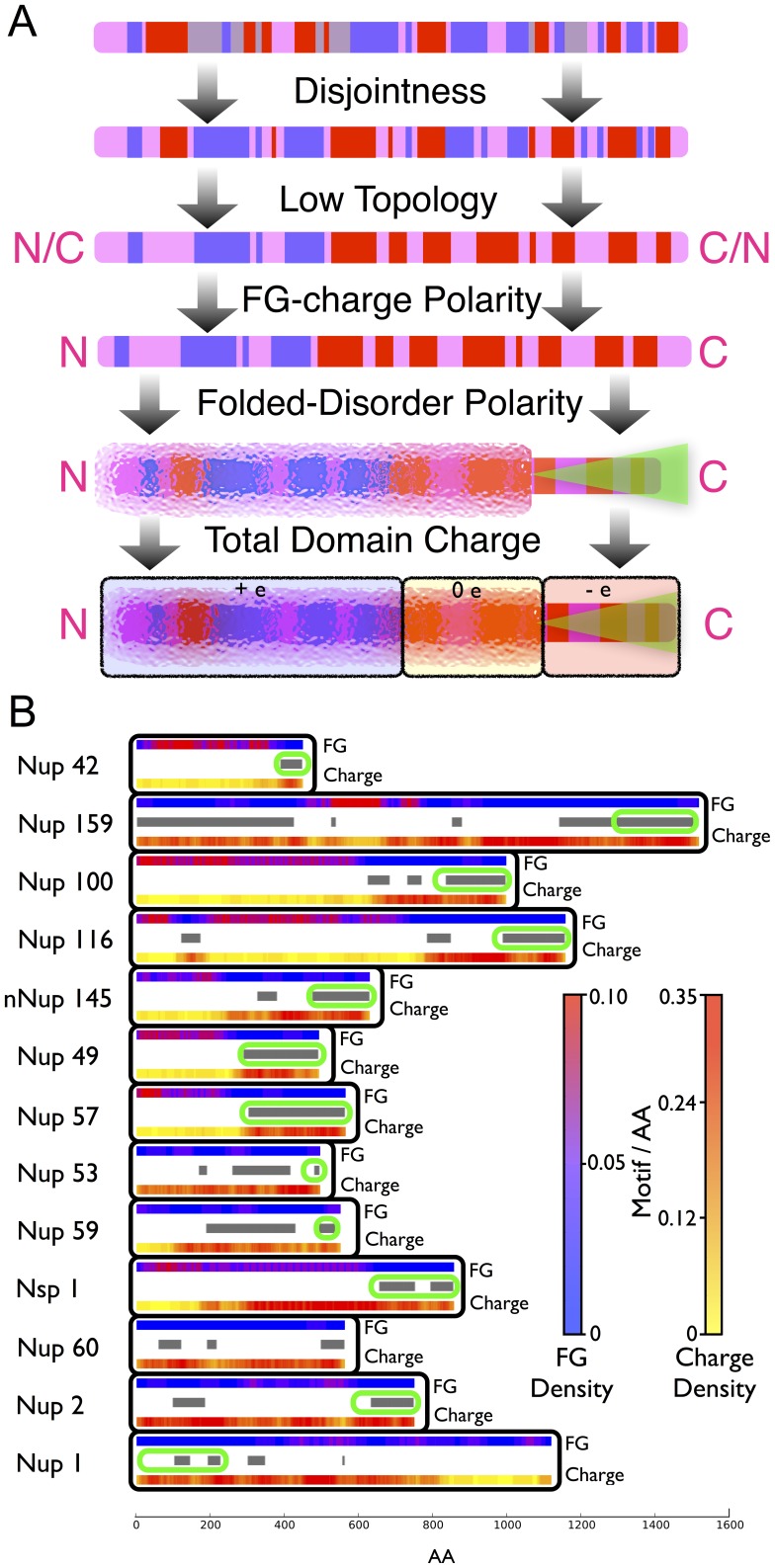
Consensus FG nup structure. (A) Effects of successively including observed sequence constraints. These constraints start from a typical randomly shuffled sequence at the very top with the pink bar representing the entire linear AA sequence. Blue blocks represent FG clusters, red blocks represent clusters of charged AAs, gray represents overlap between cluster types, green shaded regions represent folded domains which anchor to the NPC wall, while disordered domains are represented by visually solvated pixelation. The arrows originating from the starting sequence represent a possible manner by which imposing the constraint of disjointness would result in a new sequence. Similarly the successive arrows represent the imposition of further constraints found in this paper, from the low topological complexity, to the FG-Charge polarity, to the Folded-Disordered polarity, to the net charge of domains, finally culminating in an average inference which is representative of FG nups. (B) The spatial distribution of FG motifs and charged AAs for all known FG nups of S. cerevisiae plotted as motif/AA, averaged over 20 nearest AAs. Regions of high FG motif density are shown in pink while regions of low charge density, also in pink, correspond spatially throughout the sequences of these nups. Regions of protein which are predicted to form folded structures by the PONDR algorithm are highlighted with grey bars, and known/predicted [16], [26] anchor domains circled with green ovals.

Figure 3A summarizes our findings by showing the effect on a FG nup sequence of successive constraints from the observed FG-charge overlap, cluster topology, charge and order-disorder polarities. The schematic at the top of Figure 3A shows the charged AA and FG motif patterns in a randomly shuffled AA sequence and the final schematic shows the typical FG nup that fits the observed sequence constraints. Figure 3B depicts the FG and charge clusters from *all* the FG nups from a single well characterized organism, *S. cerevisiae*. The representative FG nup depicted in Figure 3A is roughly similar to most of the FG nups present in *S. cerevisiae*. The deviations of individual nups from the representative distribution and from each other provide a means via which specific FG nups could adopt distinct functional roles within the NPC.

### Functional Groupings of FG Nups

The fact that the spatial patterns of charged AAs and FG regions in FG nups which we have found, summarized in Figure 3A, are strongly conserved across eukarya provides evidence that these patterns are constrained by FG nup function. This suggests that the observed spatial patterns could provide templates for categorizing different FG nups into structural and functional subclasses. To test this, we constructed a five dimensional space determined by the biophysical metrics of FG-Charge cluster overlap, FG-Charge cluster polarity, Folded-Disordered region polarity, percent of disordered region composed of charged AA clusters, and topological complexity. Every FG nup analyzed in this paper is therefore a point in this space. We then used the density in this space to cluster these points into distinct groups. Clustering in this case is therefore not on motifs in an FG nup’s amino acid sequence (as in the earlier part of the paper) but across sets of different proteins.

**Figure 4:**
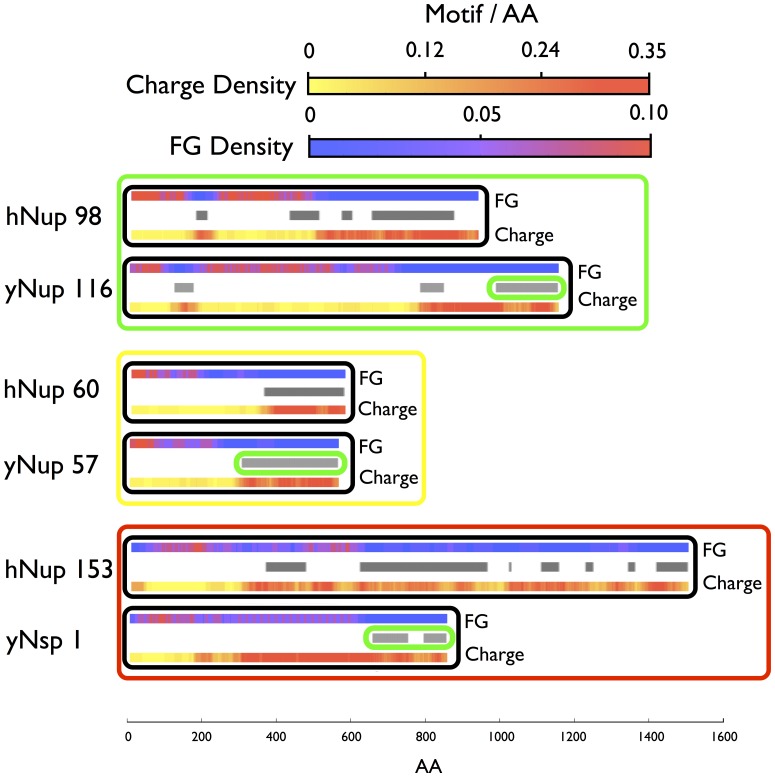
Identification of homologous proteins. Example proteins from *S. cerevisiae* (‘y’ prefix) and humans (‘h’ prefix) for each functional clustering group. Boxed in green are example proteins with low overlap between FG and charge clusters and a topological complexity of 3. In the yellow box are example proteins with low toplogical complexity and diblock structure, the first clustering group described in the text. Example proteins from the last clustering group described in the text are boxed in red at the bottom, with these proteins displaying high overlap and high levels of topological complexity (Nup153 sequence reversed to ease comparison with Nsp1).

This clustering resulted in 4 distinct groups (Text S1, Figure S8, Figure S9, Figure S10, Figure S11, Figure S12). The first group has a topological complexity of 1, a diblock structure, with a high level of disjointness and has no high density charged AA region in its disordered region. This group contains roughly one third of the FG nups with well-known FG nups in this group for *S. cerevisiae* being Nup57, and in humans Nup60 (Figure 4, Text S1, Figure S13, Figure S14).

The second group is quite small at around 5% of FG nups and consists of those proteins with a topological complexity of 2, high disjointness and no other considerable properties. Given this group’s small size and low occurrence among species, we concluded that proteins from this group are essentially outliers. On the other hand the third group is significantly sized containing approximately one quarter of FG nups with a topological complexity of 3. This group is notable for having large high density charged AA regions in its disordered regions, high disjointness and substantial negative polarities for both FG-charged AAs and folded-disordered regions. These are represented by FG nups such as Nup116 in *S. cerevisiae* and Nup98 in humans (Figure 4, Text S1, Figure S15, Figure S16).

The fourth group which represents roughly one third of FG nups tends to have high degrees of topological complexity, large charged AA disordered regions, low disjointness, and low degrees of any polarity. Examples in this group include Nsp1 in *S. cerevisiae* and Nup153 in humans (Figure 4, Text S1, Figure S17, Figure S18).

Based on the identity of S. cerevisiae proteins in each clustering group, these groups can be related to categories of FG nups previously identified in *S. cerevisiae* by Yamada *et al* [26]. Using a combination of biophysical and molecular simulation data they classified all S. cerevisiae FG nups as either compact structures (“shrubs”) or extended structures (“tree”), with the latter usually exhibiting separate regions enriched in charged AAs or FG motif sequences. Our clustering results find a similar subdivision, with cluster one corresponding to the “shrubs”, and clusters three and four corresponding to different subclasses of “trees”.

## Discussion

Regulation of nucleocytoplasmic transport by the NPC is governed by a permeability barrier whose structure, dynamics, and manner of interaction with transport factors remain elusive. Given that FG nups are a critical component of the NPC selective transport machinery [32], the conservation of observed FG motif and charge cluster arrangements over highly-divergent eukaryotic species suggest that the functional constraints of the NPC are leading to the observed patterns. These constraints could be at the level of the individual FG nups or to preserve essential interaction patterns between the FG nups.

At the individual protein level, any requirement for cooperative binding of FG motifs with transport factors (intra-strand cooperativity) could drive clustering but not necessarily the localization of FG motifs to the tips of nups. Inter-strand cooperativity between individual nups could, however, drive the localization of motifs to the tips of FG nups, because the cylindrical geometry [3] would require the tips to be in closer proximity (Figure 3B) [26]. Certain “fly casting” [11], [33], [34] mechanisms, where transport can potentially be driven by the action of single nups could also provide for a similar selective pressure at the individual FG nup level. Analogous mechanisms could be responsible for the evolutionary selection for spatial localization of positive charges to the tips of nups coincident with FG clusters, which would enhance binding with negatively-charged kaps. Depending on the geometric configuration this enhancement could be substantially above the levels proposed in [31] where charge localization was not considered.

At the collective level, the interactions between the large number of FG nups in the NPC have been hypothesized to lead to polymer brushes, hydrogels, reduced dimensionality surfaces, and other more complex structures, such as the transporter/plug structure. These emergent structures and their dynamics may require spatially-separated charge and FG clusters for numerous reasons. There have been a number of experimental observations which suggest FG nups interact to form larger scale emergent structures, including observations of a transporter structure [35], modern non-invasive FESE microscopy imaging of a central particle [36], AFM imaging of a central bump [37], and cargo specific spatial pathways through the NPC [38], [39].

Another element in FG nup sequence organization that offers clues to NPC function is that their charged disordered regions contain a large fraction of charged AAs (about 1/3 on average) yet are nearly neutral as a whole. This is unusual for disordered domains [40]. This selective pressure for net neutrality while containing a high density of charged residues, raises the possibility that electrostatic interactions could be a key part of the overall selective mechanism for NPC transport [41]. Current models of NPC transport [42], [43] typically involve homogeneous nups and are not based on any particular spatial distribution of FG motifs and charged residues, nor have any functional or structural need for entropic-chain domains with enhanced charge densities. Our findings provide a new set of evolutionarily conserved properties of the FG nups that need to be explained by proposed NPC transport models. More broadly, our approach of focusing on the abstract arrangement of physico-chemical features within sequences rather than the specific AA sequence shows promise in allowing for the identification of conserved features that share functionality across different types of proteins, but especially among functionally-equivalent disordered proteins with highly-disparate AA sequences.

In summary, our results argue that FG nup functionality is mediated via the arrangement of coarse-grained biophysical properties along the protein length, rather than by a precise sequence of specific AAs. This insight allowed us to identify templates that group FG nups into functional categories across widely different species despite their low sequence similarities. Moreover, the existence of these functional groups within the NPC of each species suggests a specialization of FG nups for different functional roles within the NPC. This approach can potentially be applied to gain functional and evolutionary insight into other classes of disordered proteins which have remained inaccessible to current bioinformatics techniques.

## Methods

Two groups of proteins were created. The first group, labeled NUP, contained proteins which were associated with the Nuclear Pore Complex via the keyword ‘nucleoporin’ in the Uniprot database (www.uniprot.org) on June 29, 2012. Proteins in NUP which were classified as fragments rather than full length protein sequences were removed. A second group of proteins, labeled CONTROL, was created by systematically searching the Uniprot database for 12 proteins which contained 8 or more WG/GW motif repeats and which had substantial disorder, defined as containing disordered regions in excess of 100 amino acids in length as determined by the PONDR-FIT 2010 protein disorder prediction algorithm. Similarly 12 non-nucleoporins were taken from *S. cerevisiae* which contained FG motif repeats with greater than 100 disordered amino acids were taken from Nguyen et al [30] and added to CONTROL. Associated to each protein in these two groups was a collection of forty protein sequences, labeled ENSEMBLE, identical in all respects except for having the locations of all individual amino acids randomly permuted via a random shuffling.

Cluster identification was first done by transforming protein amino acid sequences into a series of binary representations with the specified motif set as unity and all other amino acids set to zero. The FG repeat containing proteins were parsed using the “FG” and “GF” motifs, WG repeats which were parsed using the “WG” and “GW” motifs, and charge motifs which were parsed together from the single AA motifs “D”, “K”, “E”, and “R”. The “GF” motif was used in addition to the “FG” motif due its chemical similarity and high degree of spatial correlation to “FG” motifs along the AA sequence of FG nups (Text S1). The PreDeCon clustering algorithm was then applied to the one dimensional binary representations with a minimum cluster size of two amino acids (one AA for each cluster boundary) and a clustering resolution epsilon which maximized the Dunn validity index [25]. Clustering was done within the ELKI data mining software package [44]. A series of additional clustering variations were considered using various levels of reduced amino acid alphabets derived from the BLOSUM50 AA similarity [45] (Text S1).

All proteins which were not members of ENSEMBLE were additionally analyzed for the locations of disordered and folded regions using the PONDR-FIT 2010 algorithm [22]. A PONDR-FIT score for an amino acid less than 0.5 indicated a propensity for this location to be a folded region and in our analysis the protein was assumed to be fold at this location while a score greater than 0.5 indicated a disordered region and the protein was assumed to be natively unfolded at this location.

The NUP group proteins were characterized by the percentage of probable disorder and by their FG motif density (FG motif repeats/AA), where two groups arose naturally when restricting NUP to proteins with greater than 400 AAs, one with low percentage disorder and FG density, and another group with relatively high percentage disorder and FG density (Text S1). We took proteins in the NUP group with greater than 0.15 FG/AA linear motif density and greater than 30% disorder to be the FG nups which we analyzed in this study.

For each FG/WG motif cluster in each protein, including the ENSEMBLE, the number of amino acids which were simultaneously in both charge clusters and the FG/WG motif cluster was counted and then divided by the number of total amino acids in the FG/WG cluster. This was the percentage overlap for clusters while the standard deviation of a nup in terms of cluster overlap measured the difference in total overlap between cluster types between a FG nup or control protein and its ENSEMBLE in standard deviations of the ENSEMBLE.

For each protein, including the ENSEMBLE, topological complexity was calculated by counting the number of times a sequence had a change in cluster type disregarding regions of the protein which did not fall within a FG/WG or charge cluster. Explicitly this was calculated by first computing a 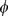 function mapping amino acid 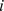 to the values 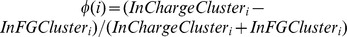 for variables 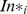 true (1) or false (0) for AA 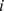 inside the indicated cluster type, omitting regions of the protein that had both 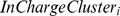 and 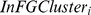 equal to zero. The topological complexity was then derived by counting the number of times that this 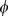 function had a non zero difference. In determining net charges of regions the only charged AAs were assumed to be D (−1), K (+1), E (−1), and R (+1) with Histidine excluded due to it being charged only 10% of the time at physiological ph [46] and because of its high depletion levels in FG nups [26]. All charge values are measured in normalized units of charge per AA length. The calculation of the net charge of folded regions of FG nups was restricted to folded regions with greater than 4 AA to avoid noise in the fold prediction algorithm. The net charge of disordered regions of FG nups which excludes FG clusters but contains charge clusters was determined by considering only the disordered regions which were mostly composed of charge clusters. With charge clusters having a charged AA density of roughly 30% we used a cutoff that required these disordered regions to have greater than 15% charged AAs.

The 1,167 FG nups analyzed (Data S1) were additionally clustered among themselves using the PreDeCon algorithm [23] over a five dimensional space spanning the properties measured in the paper (Text S1). Setting the minimum cluster size to 50 proteins with a unitary neighborhood allowed for a large scale overview of how the 1,167 FG nups organize themselves in this five dimensional space, which resulted in four major groupings of FG nups.

## Supporting Information

Figure S1 **Histogram of percentage overlap for generalized and simplified FG motifs (LVIMCAGSTPFYW, FYW, and F) versus various characterizations of polar amino acids (EDNQKRH, EDKR, and K).** Analyzing FG nups using FG motif versus charged AAs results in a clustering with the least degree of overlap, and is therefore the simplest and easiest to interpret.
(TIFF)

Figure S2 **Continuous distribution of nups across NUP in terms of percent disorder (y-axis) and FG density in FG motifs/AA (x-axis).**
(TIFF)

Figure S3 **Natural split for nups for NUP restricted to proteins with greater than 400 AA at roughly greater than 10% FG/AA FG motif density and greater than 30% protein disorder.**
(TIFF)

Figure S4 **Natural split for Bakers Yeast, with 400 AA restriction.** Yellow circle highlights refer to known FG nups while grey dots which are not highlighted represent known structural/transport proteins.
(TIFF)

Figure S5 **Natural split for humans, with 400 AA restriction.** Yellow circle highlights refer to known FG nups while grey dots which are not highlighted represent known structural/transport proteins.
(TIFF)

Figure S6 **Net charge/AA for charged disordered charged regions of FG nups.** The net charge for disordered charged regions of FG nups histogram is symmetric around zero net charge indicating that on average these regions are not selected for any net charge and on average are charge neutral.
(TIFF)

Figure S7 **Net charge/AA for folded regions of FG nups is symmetric around zero net charge indicating that on average these regions are not selected for any net charge and on average are neutrally charged.**
(TIFF)

Figure S8 **Histogram of FG-charge cluster percentage overlap in FG nups.** Density clustering separates FG nups into 4 distinct groups, which are labeled, yellow, green, red and blue. Along the disjointness axis (x-axis) the red, green, and blue cluster groups aggregate at very high levels of disjointness, while the red group tends to have moderate levels of overlap.
(TIFF)

Figure S9 **Histogram of FG-Charge cluster polarity.** Density clustering separates FG nups into 4 distinct groups, which are shown in yellow, green, red and blue. Along the FG-Charge polarity axis positive polarity nups are nearly entirely from the red group, while other groups have significant negative polarity.
(TIFF)

Figure S10 **Histogram of FG nup Folded-Disordered region polarity.** Density clustering separates FG nups into 4 distinct groups, which are labeled, yellow, green, red and blue. The positive polarity nups are nearly entirely from the red group, while other groups have significant negative polarity.
(TIFF)

Figure S11 **Histogram of the percent of disordered region composed of charged AA clusters for FG nups.** Density clustering separates FG nups into 4 distinct groups, shown in yellow, green, red and blue. Along the percentage charged cluster axis the yellow group few charged amino acid clusters in the disordered regions, while the other three groups have significant charged disordered regions.
(TIFF)

Figure S12 **Histogram of the topological complexity of FG nups.** Density clustering separates FG nups into 4 distinct groups, shown in yellow, green, red and blue. Along the topological complexity axis the yellow group has exclusively a topological complexity of 1, the blue group a topological complexity of 2, the green group a topological complexity of 3, while the red group has a topological complexity distributed over a range of higher values.
(TIFF)

Figure S13 **FG nucleoporin from S. cerevisiae, Nup49, from the yellow group.** Amino acid sequence number is shown along the x-axis for all sub-charts. The first chart starting from the top shows QN amino acids as red vertical lines and their clusters colored alternately blue and green as a control. Similarly colored is the second chart which shows charged amino acids, while the third chart has FG motifs represented as red vertical lines with clusters represented by alternating purple and cyan regions. The fourth chart displays the propensity for protein disorder for a given AA as predicted by PONDR, with red representing high propensity and yellow representing low propensity. Green circles represent centers of masses of cluster regions and the purple arrow indicates disordered region to folded region polarity.
(TIFF)

Figure S14 **FG nucleoporin from Homo sapiens, Nup62, from the yellow group.** Amino acid sequence number is shown along the x-axis for all sub-charts. The first chart starting from the top shows QN amino acids as red vertical lines and their clusters colored alternately blue and green as a control. Similarly colored is the second chart which shows charged amino acids, while the third chart has FG motifs represented as red vertical lines with clusters represented by alternating purple and cyan regions. The fourth chart displays the propensity for protein disorder for a given AA as predicted by PONDR, with red representing high propensity and yellow representing low propensity. Green circles represent centers of masses of cluster regions and the purple arrow indicates disordered region to folded region polarity.
(TIFF)

Figure S15 **FG nucleoporin from S. cerevisiae, Nup116, from the green group.** Amino acid sequence number is shown along the x-axis for all sub-charts. The first chart starting from the top shows QN amino acids as red vertical lines and their clusters colored alternately blue and green as a control. Similarly colored is the second chart which shows charged amino acids, while the third chart has FG motifs represented as red vertical lines with clusters represented by alternating purple and cyan regions. The fourth chart displays the propensity for protein disorder for a given AA as predicted by PONDR, with red representing high propensity and yellow representing low propensity. Green circles represent centers of masses of cluster regions and the purple arrow indicates disordered region to folded region polarity.
(TIFF)

Figure S16 **FG nucleoporin from Homo sapiens, Nup98, from the green group.** Amino acid sequence number is shown along the x-axis for all sub-charts. The first chart starting from the top shows QN amino acids as red vertical lines and their clusters colored alternately blue and green as a control. Similarly colored is the second chart which shows charged amino acids, while the third chart has FG motifs represented as red vertical lines with clusters represented by alternating purple and cyan regions. The fourth chart displays the propensity for protein disorder for a given AA as predicted by PONDR, with red representing high propensity and yellow representing low propensity. Green circles represent centers of masses of cluster regions and the purple arrow indicates disordered region to folded region polarity.
(TIFF)

Figure S17 **FG nucleoporin from S. cerevisiae, Nsp1, from the red group.** Amino acid sequence number is shown along the x-axis for all sub-charts. The first chart starting from the top shows QN amino acids as red vertical lines and their clusters colored alternately blue and green as a control. Similarly colored is the second chart which shows charged amino acids, while the third chart has FG motifs represented as red vertical lines with clusters represented by alternating purple and cyan regions. The fourth chart displays the propensity for protein disorder for a given AA as predicted by PONDR, with red representing high propensity and yellow representing low propensity. Green circles represent centers of masses of cluster regions and the purple arrow indicates disordered region to folded region polarity.
(TIFF)

Figure S18 **FG nucleoporin from Homo sapiens, Nup153, from the red group.** Amino acid sequence number is shown along the x-axis for all sub-charts. The first chart starting from the top shows QN amino acids as red vertical lines and their clusters colored alternately blue and green as a control. Similarly colored is the second chart which shows charged amino acids, while the third chart has FG motifs represented as red vertical lines with clusters represented by alternating purple and cyan regions. The fourth chart displays the propensity for protein disorder for a given AA as predicted by PONDR, with red representing high propensity and yellow representing low propensity. Green circles represent centers of masses of cluster regions and the purple arrow indicates disordered region to folded region polarity.
(TIFF)

Dataset S1 **Supporting dataset.**
(TXT)

Text S1 **Supporting text.**
(PDF)
