# Supplementary figures and images for: Physical Motif Clustering within Intrinsically Disordered Nucleoporin Sequences Reveals Universal Functional Features
Source: PLoS One. 2013 Sep 16;8(9):e73831. doi: 10.1371/journal.pone.0073831 (PMC3774778; doi:10.1371/journal.pone.0073831)

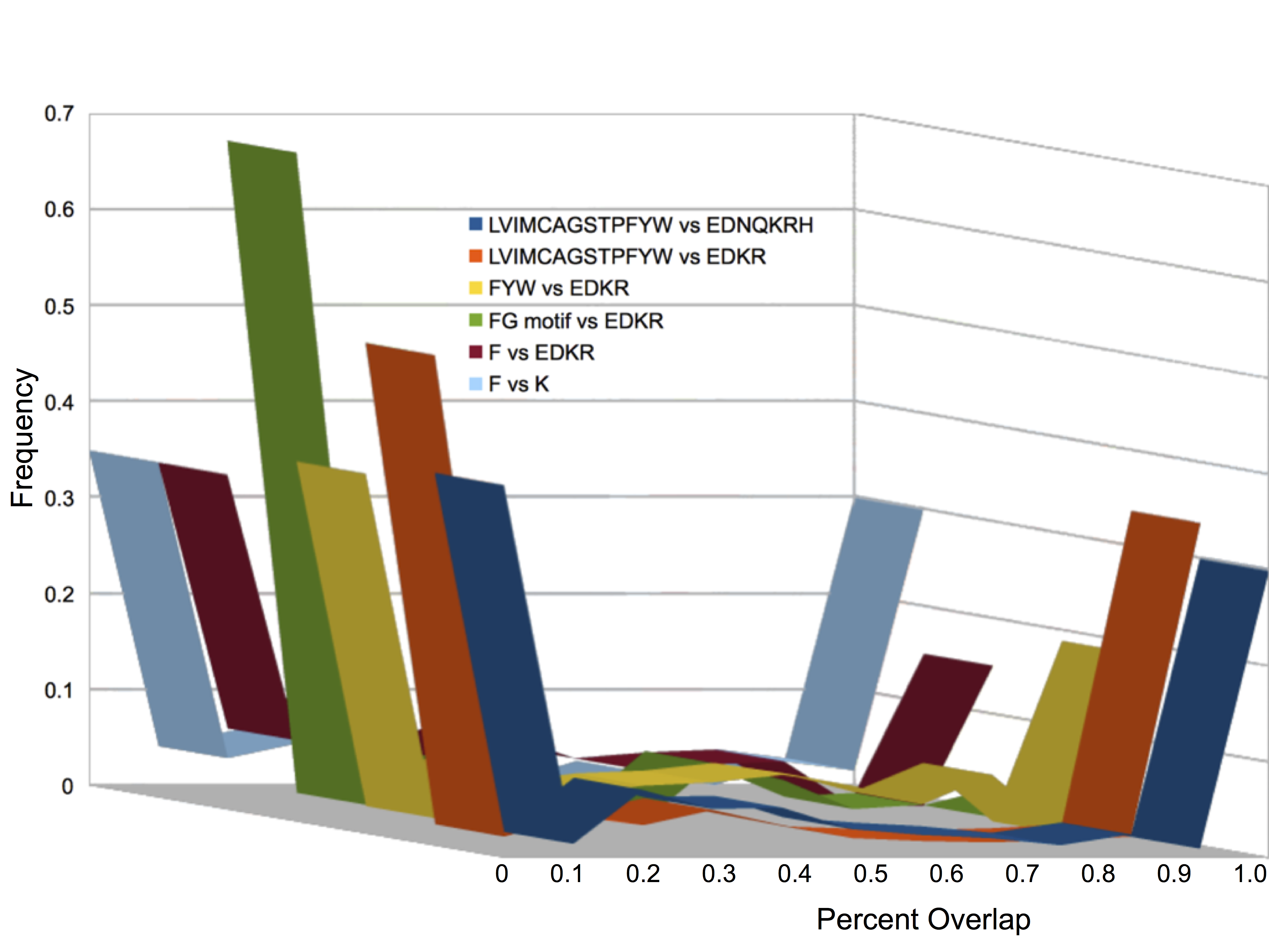

Supplement: Figure S1 — Histogram of percentage overlap for generalized and simplified FG motifs (LVIMCAGSTPFYW, FYW, and F) versus various characterizations of polar amino acids (EDNQKRH, EDKR, and K). Analyzing FG nups using FG motif versus charged AAs results in a clustering with the least degree of overlap, and is therefore the simplest and easiest to interpret. (TIFF) [file pone.0073831.s001.tiff]

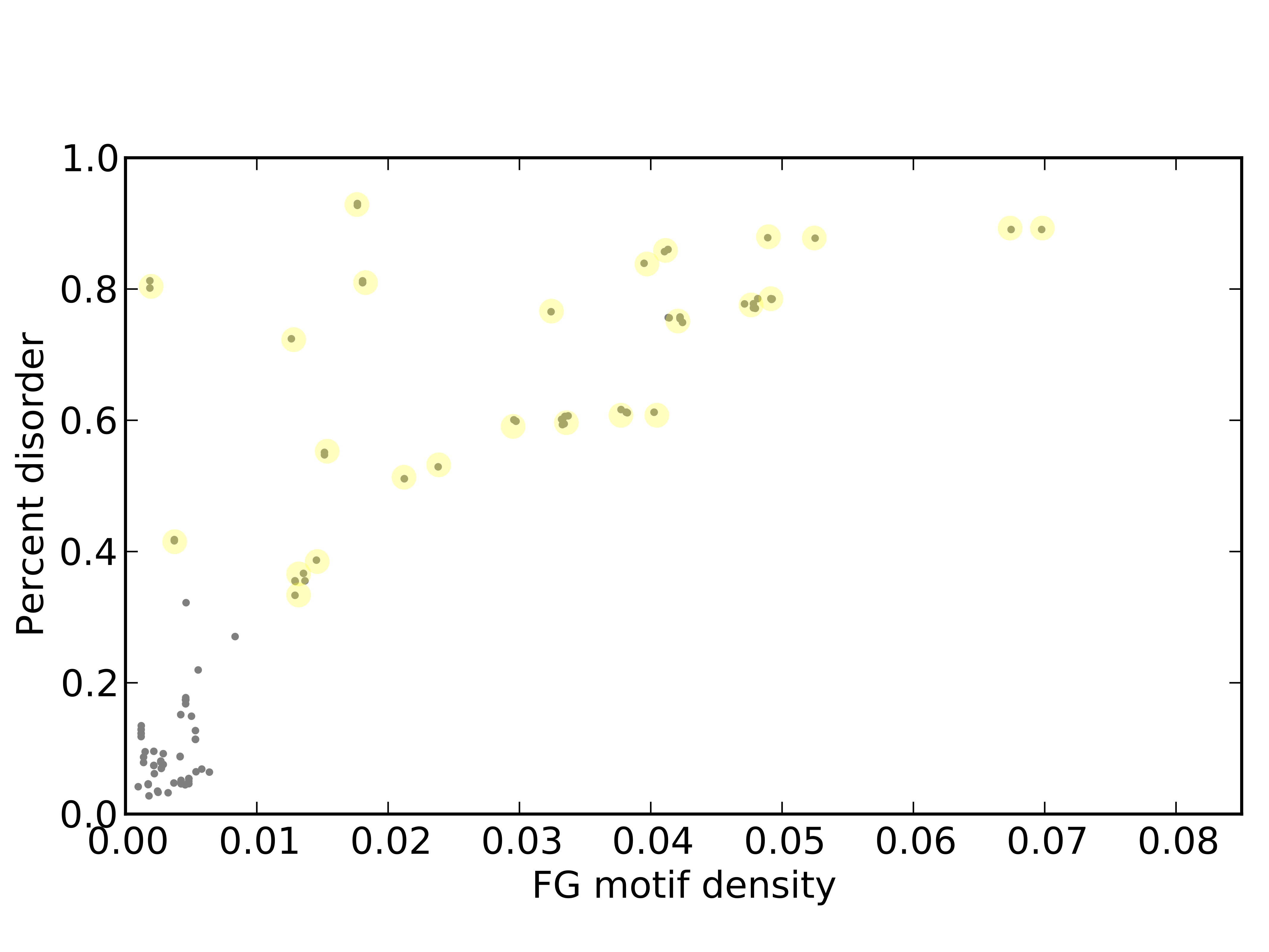

Supplement: Figure S4 — Natural split for Bakers Yeast, with 400 AA restriction. Yellow circle highlights refer to known FG nups while grey dots which are not highlighted represent known structural/transport proteins. (TIFF) [file pone.0073831.s004.tiff]

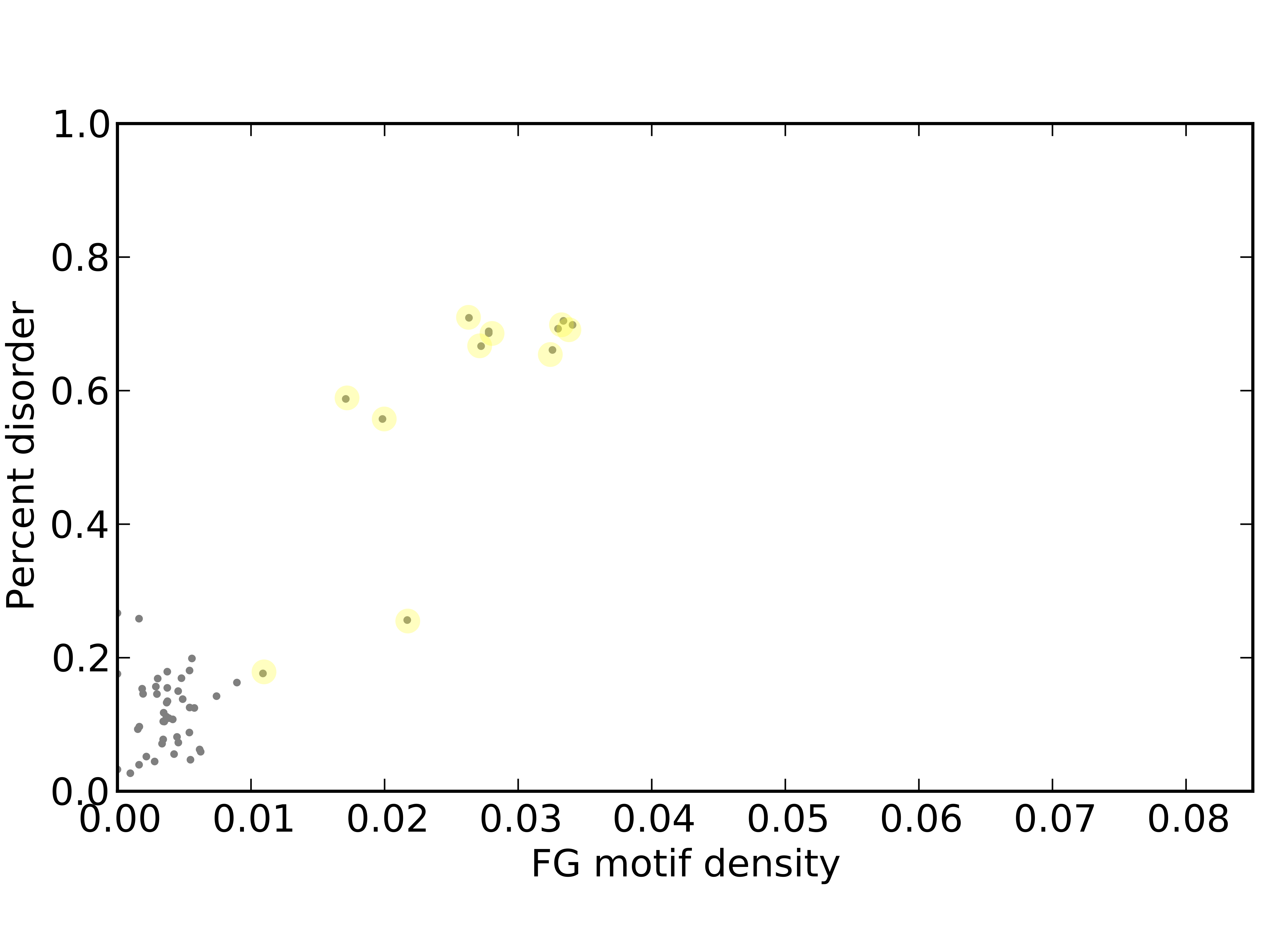

Supplement: Figure S5 — Natural split for humans, with 400 AA restriction. Yellow circle highlights refer to known FG nups while grey dots which are not highlighted represent known structural/transport proteins. (TIFF) [file pone.0073831.s005.tiff]

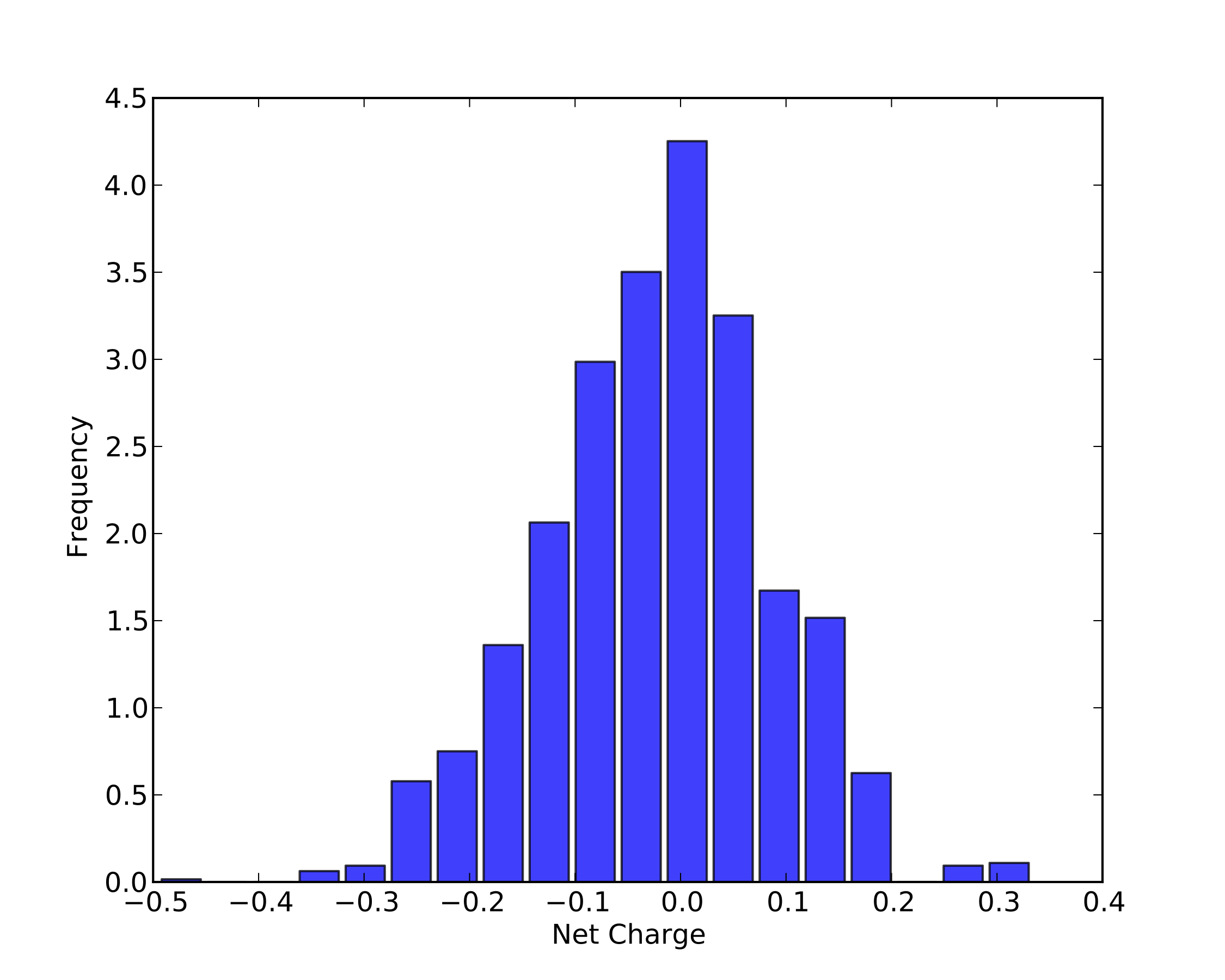

Supplement: Figure S6 — Net charge/AA for charged disordered charged regions of FG nups. The net charge for disordered charged regions of FG nups histogram is symmetric around zero net charge indicating that on average these regions are not selected for any net charge and on average are charge neutral. (TIFF) [file pone.0073831.s006.tiff]

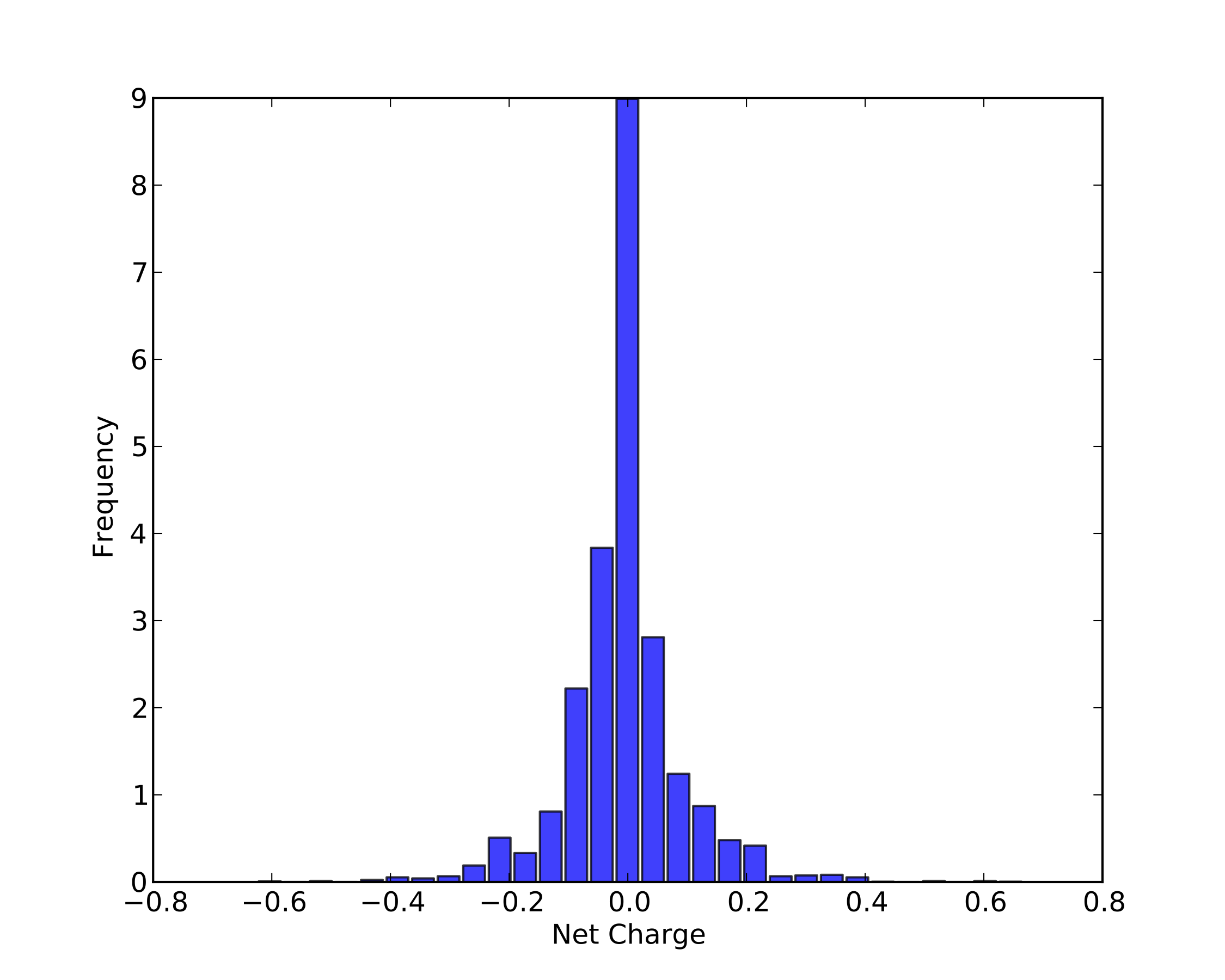

Supplement: Figure S7 — Net charge/AA for folded regions of FG nups is symmetric around zero net charge indicating that on average these regions are not selected for any net charge and on average are neutrally charged. (TIFF) [file pone.0073831.s007.tiff]

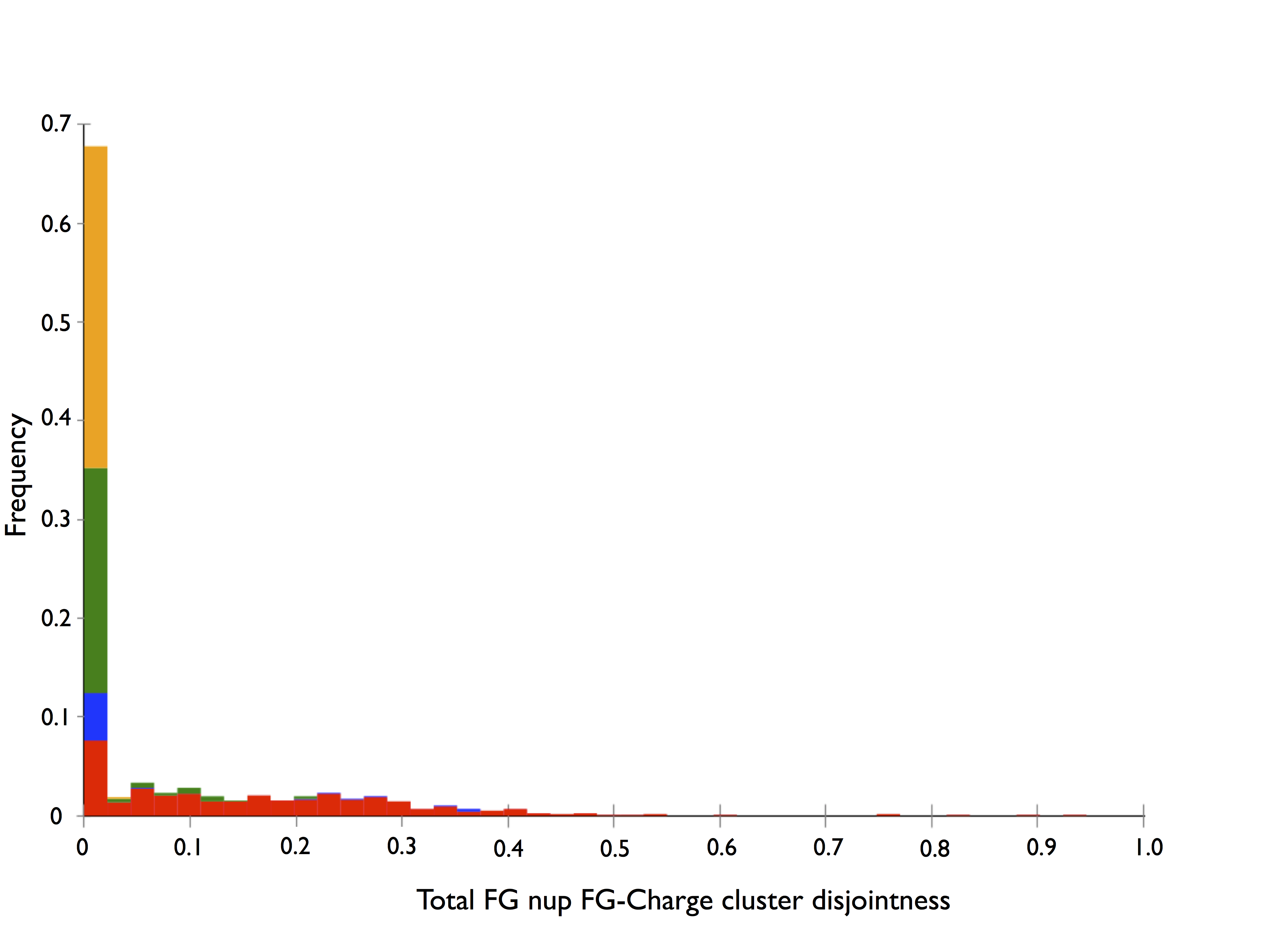

Supplement: Figure S8 — Histogram of FG-charge cluster percentage overlap in FG nups. Density clustering separates FG nups into 4 distinct groups, which are labeled, yellow, green, red and blue. Along the disjointness axis (x-axis) the red, green, and blue cluster groups aggregate at very high levels of disjointness, while the red group tends to have moderate levels of overlap. (TIFF) [file pone.0073831.s008.tiff]

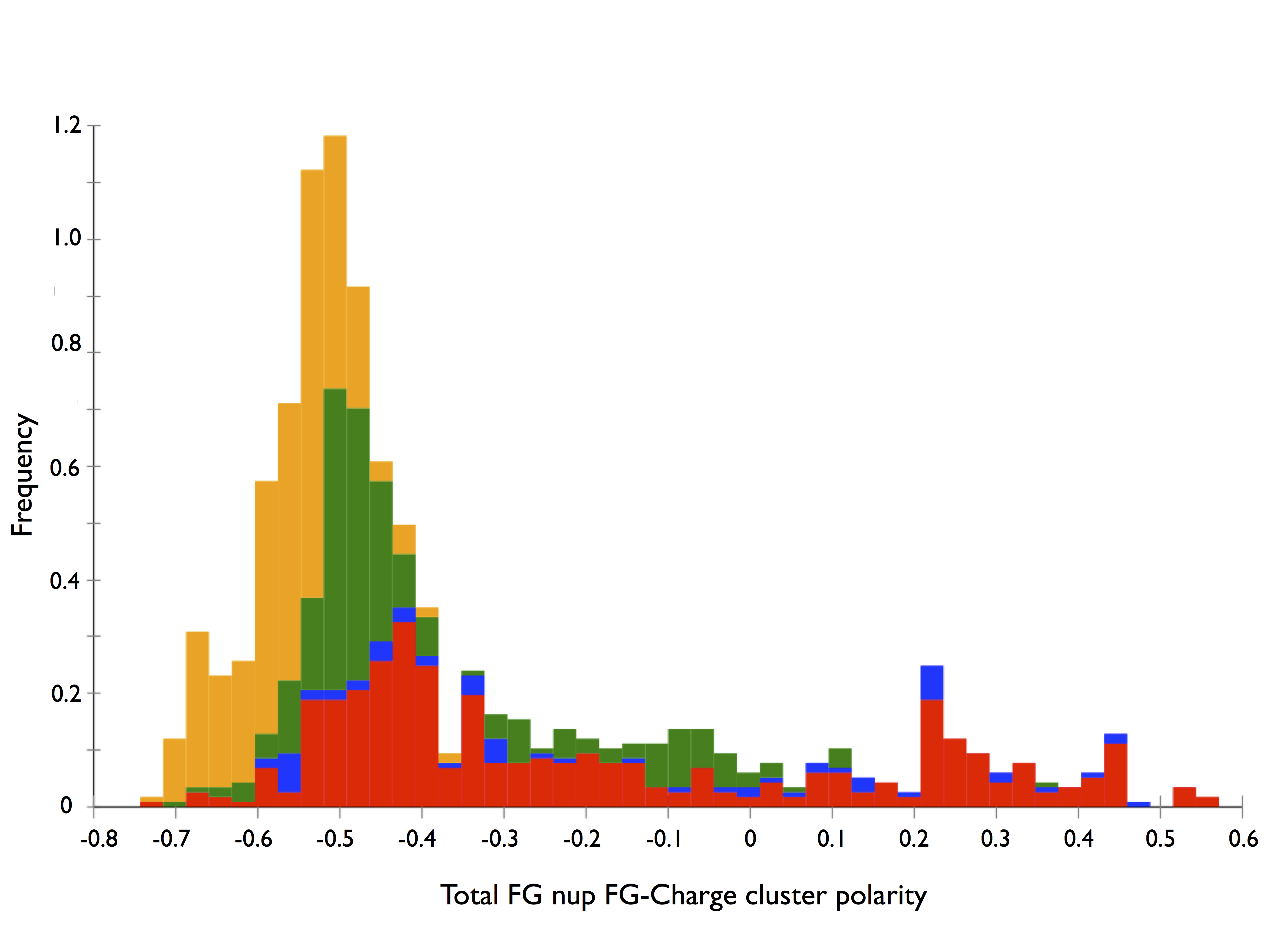

Supplement: Figure S9 — Histogram of FG-Charge cluster polarity. Density clustering separates FG nups into 4 distinct groups, which are shown in yellow, green, red and blue. Along the FG-Charge polarity axis positive polarity nups are nearly entirely from the red group, while other groups have significant negative polarity. (TIFF) [file pone.0073831.s009.tiff]

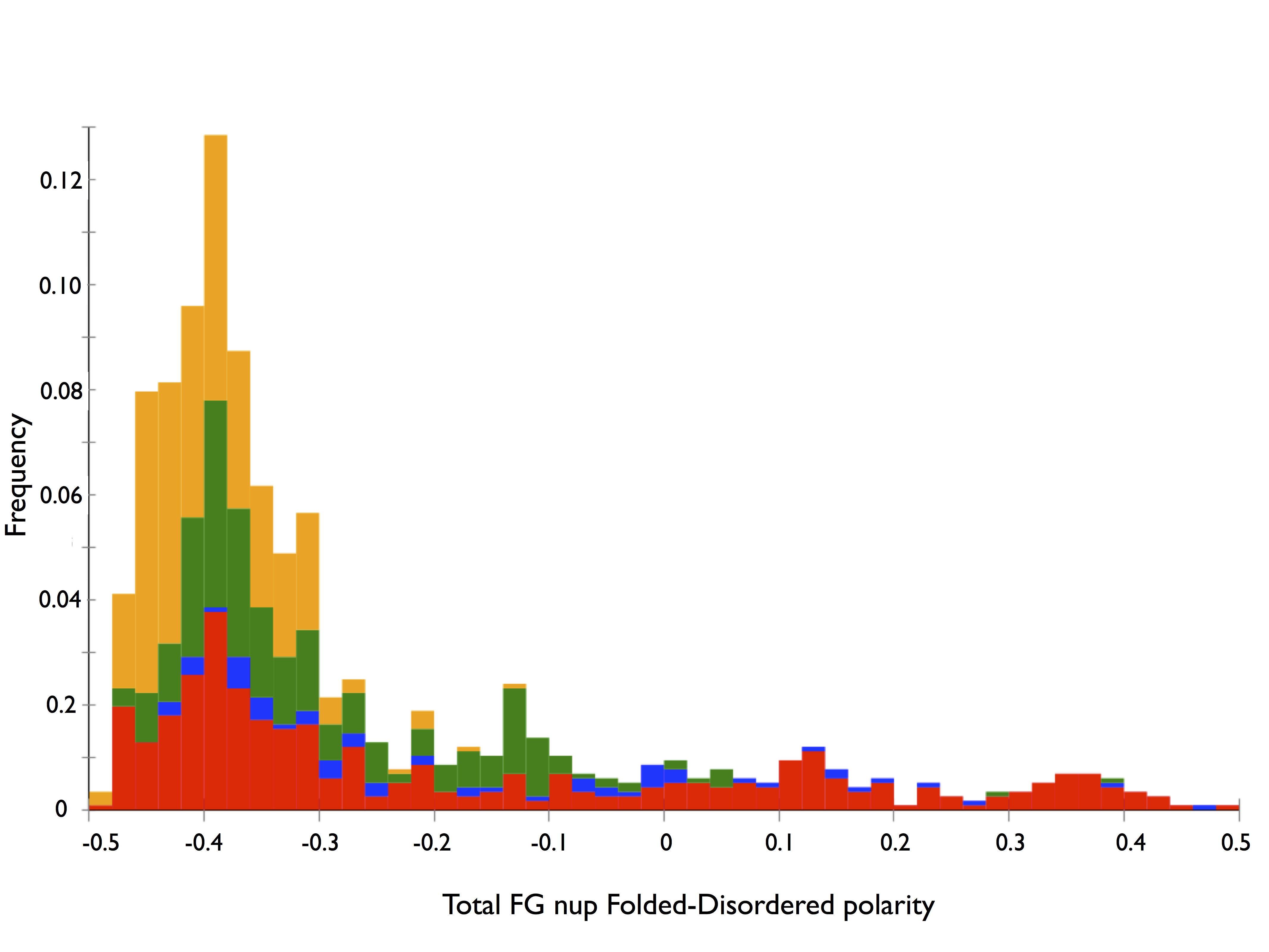

Supplement: Figure S10 — Histogram of FG nup Folded-Disordered region polarity. Density clustering separates FG nups into 4 distinct groups, which are labeled, yellow, green, red and blue. The positive polarity nups are nearly entirely from the red group, while other groups have significant negative polarity. (TIFF) [file pone.0073831.s010.tiff]

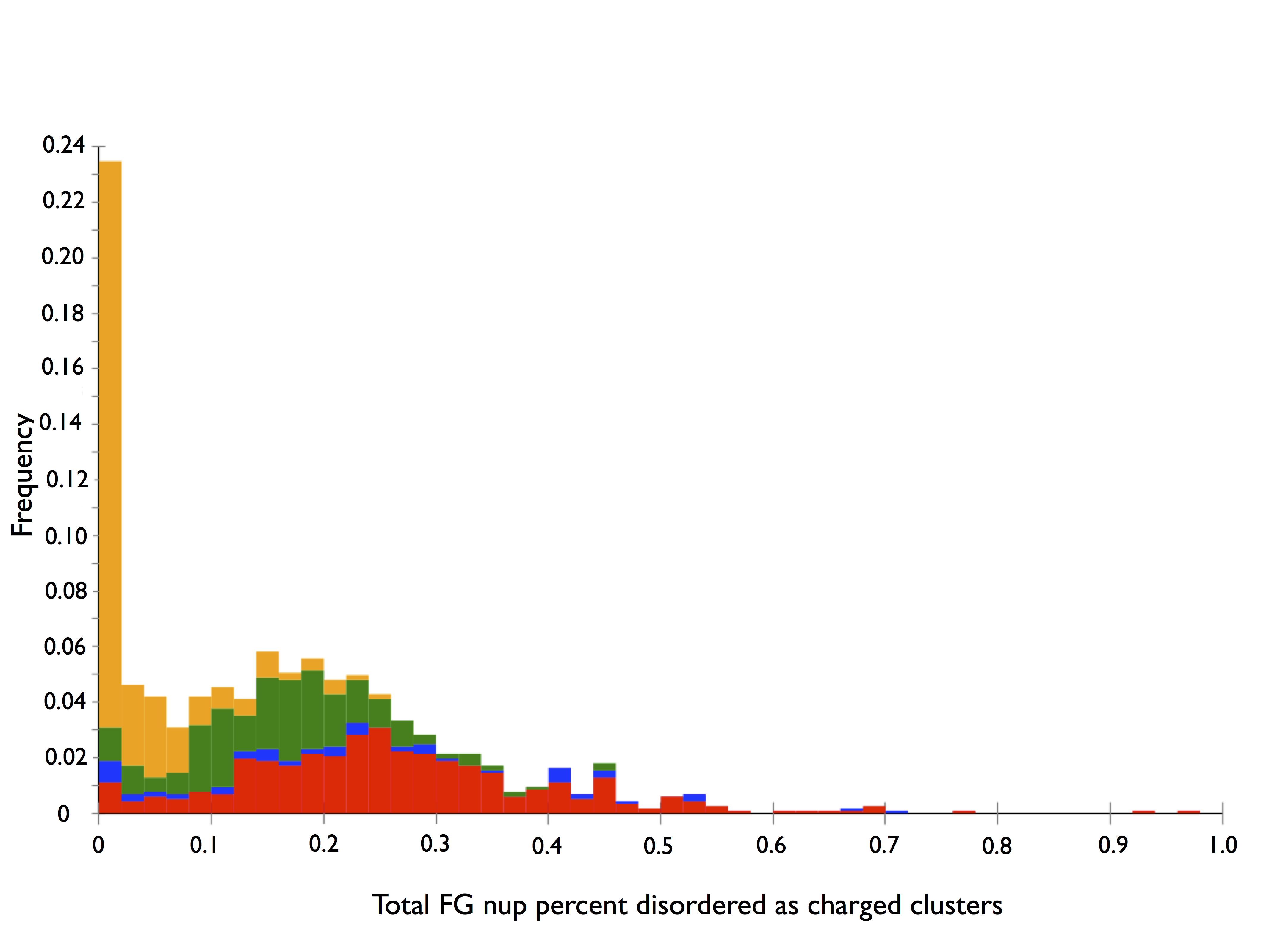

Supplement: Figure S11 — Histogram of the percent of disordered region composed of charged AA clusters for FG nups. Density clustering separates FG nups into 4 distinct groups, shown in yellow, green, red and blue. Along the percentage charged cluster axis the yellow group few charged amino acid clusters in the disordered regions, while the other three groups have significant charged disordered regions. (TIFF) [file pone.0073831.s011.tiff]

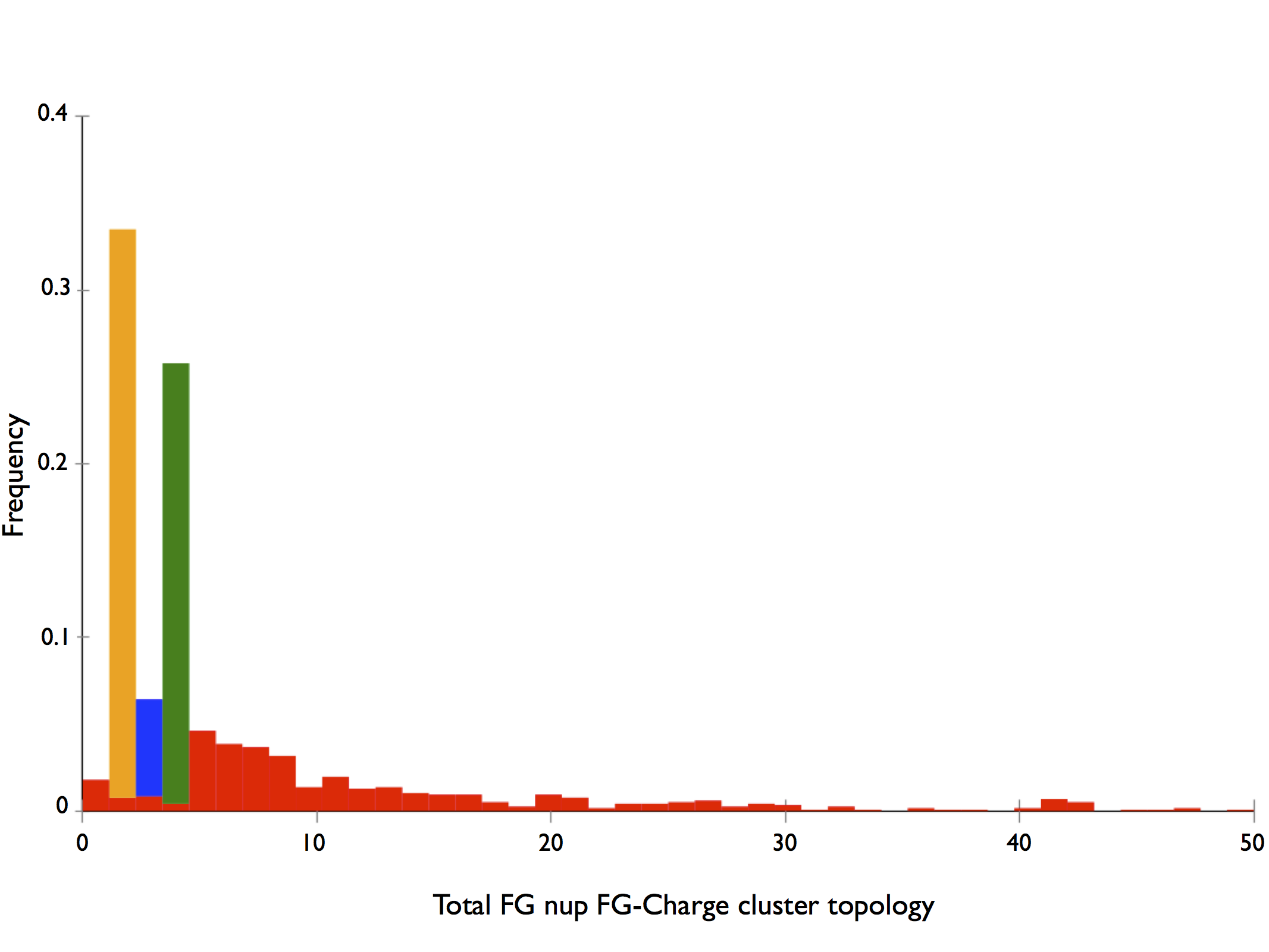

Supplement: Figure S12 — Histogram of the topological complexity of FG nups. Density clustering separates FG nups into 4 distinct groups, shown in yellow, green, red and blue. Along the topological complexity axis the yellow group has exclusively a topological complexity of 1, the blue group a topological complexity of 2, the green group a topological complexity of 3, while the red group has a topological complexity distributed over a range of higher values. (TIFF) [file pone.0073831.s012.tiff]

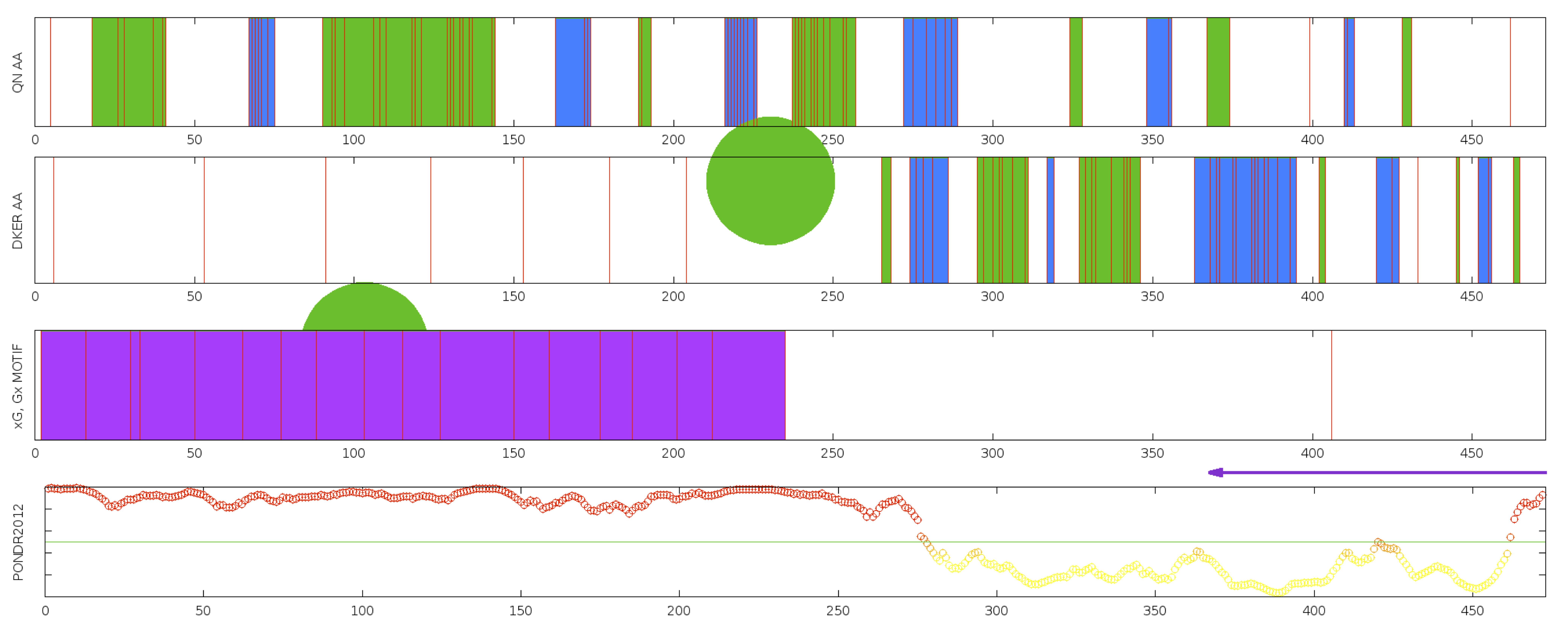

Supplement: Figure S13 — FG nucleoporin from S. cerevisiae, Nup49, from the yellow group. Amino acid sequence number is shown along the x-axis for all sub-charts. The first chart starting from the top shows QN amino acids as red vertical lines and their clusters colored alternately blue and green as a control. Similarly colored is the second chart which shows charged amino acids, while the third chart has FG motifs represented as red vertical lines with clusters represented by alternating purple and cyan regions. The fourth chart displays the propensity for protein disorder for a given AA as predicted by PONDR, with red representing high propensity and yellow representing low propensity. Green circles represent centers of masses of cluster regions and the purple arrow indicates disordered region to folded region polarity. (TIFF) [file pone.0073831.s013.tiff]

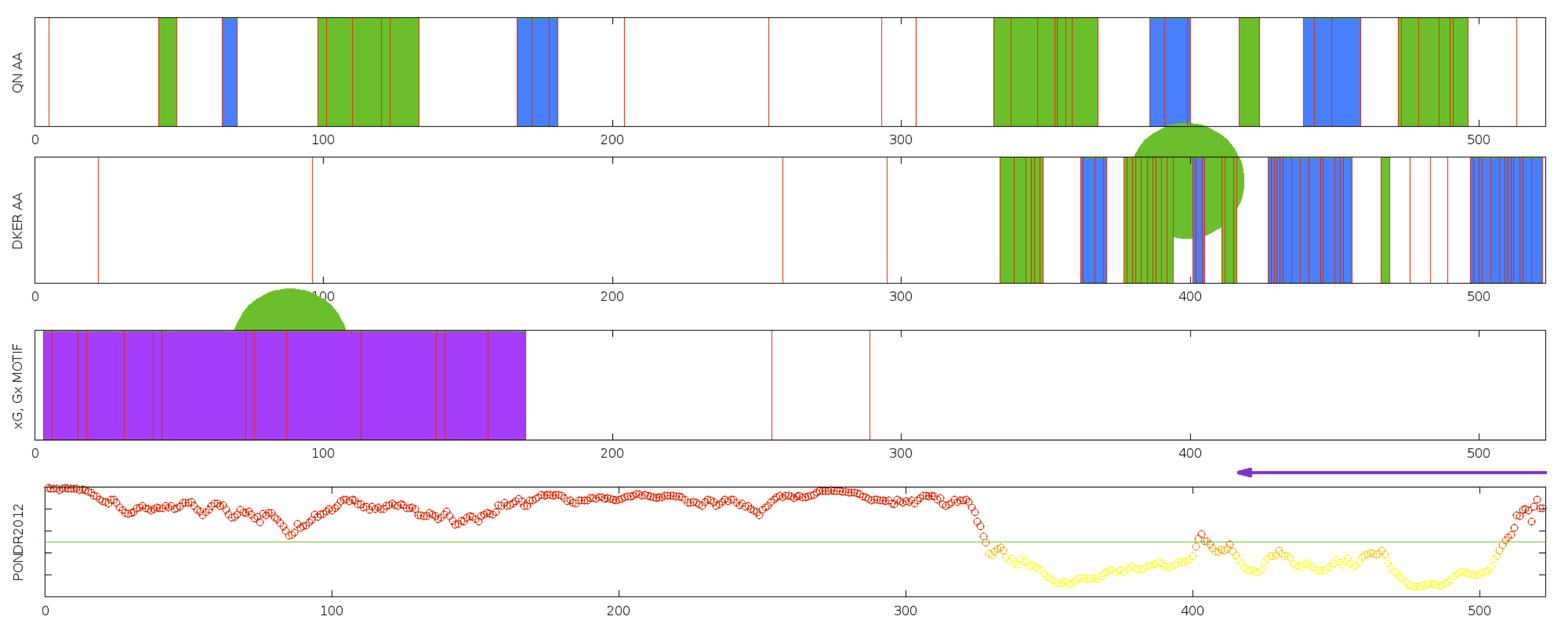

Supplement: Figure S14 — FG nucleoporin from Homo sapiens, Nup62, from the yellow group. Amino acid sequence number is shown along the x-axis for all sub-charts. The first chart starting from the top shows QN amino acids as red vertical lines and their clusters colored alternately blue and green as a control. Similarly colored is the second chart which shows charged amino acids, while the third chart has FG motifs represented as red vertical lines with clusters represented by alternating purple and cyan regions. The fourth chart displays the propensity for protein disorder for a given AA as predicted by PONDR, with red representing high propensity and yellow representing low propensity. Green circles represent centers of masses of cluster regions and the purple arrow indicates disordered region to folded region polarity. (TIFF) [file pone.0073831.s014.tiff]

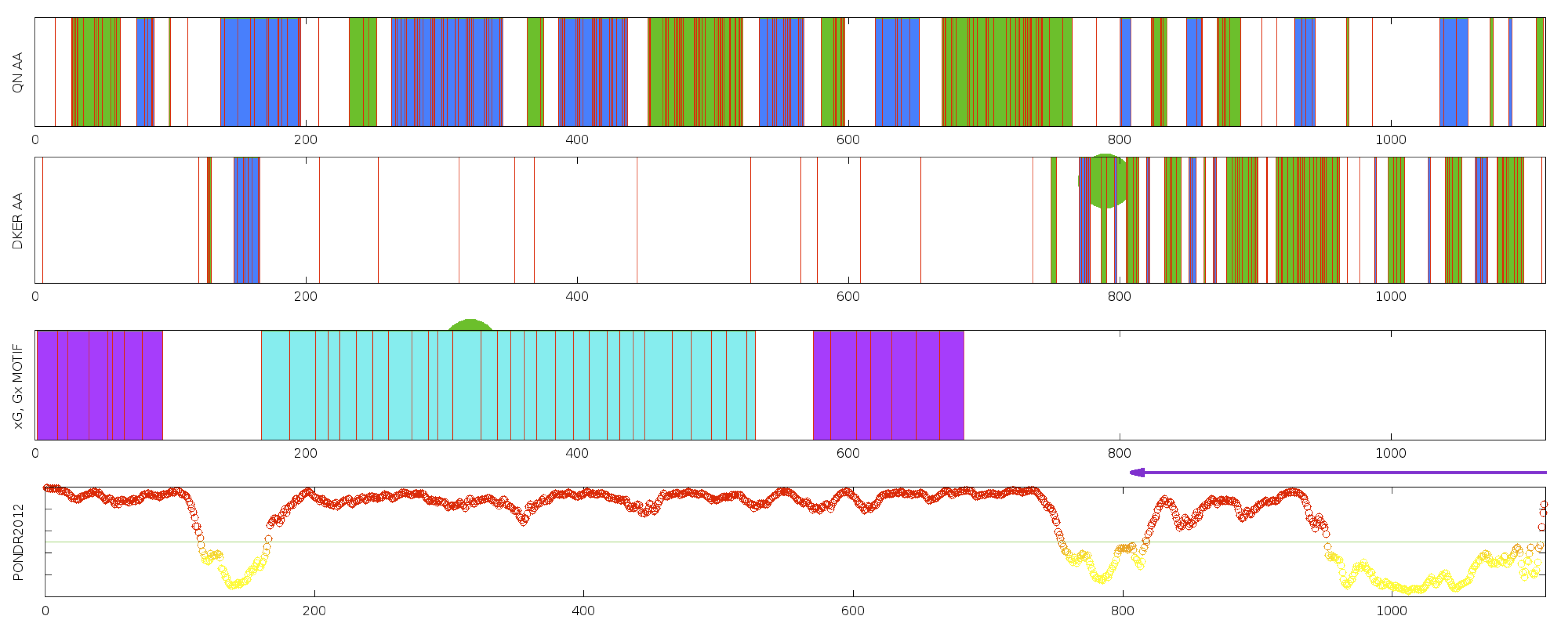

Supplement: Figure S15 — FG nucleoporin from S. cerevisiae, Nup116, from the green group. Amino acid sequence number is shown along the x-axis for all sub-charts. The first chart starting from the top shows QN amino acids as red vertical lines and their clusters colored alternately blue and green as a control. Similarly colored is the second chart which shows charged amino acids, while the third chart has FG motifs represented as red vertical lines with clusters represented by alternating purple and cyan regions. The fourth chart displays the propensity for protein disorder for a given AA as predicted by PONDR, with red representing high propensity and yellow representing low propensity. Green circles represent centers of masses of cluster regions and the purple arrow indicates disordered region to folded region polarity. (TIFF) [file pone.0073831.s015.tiff]

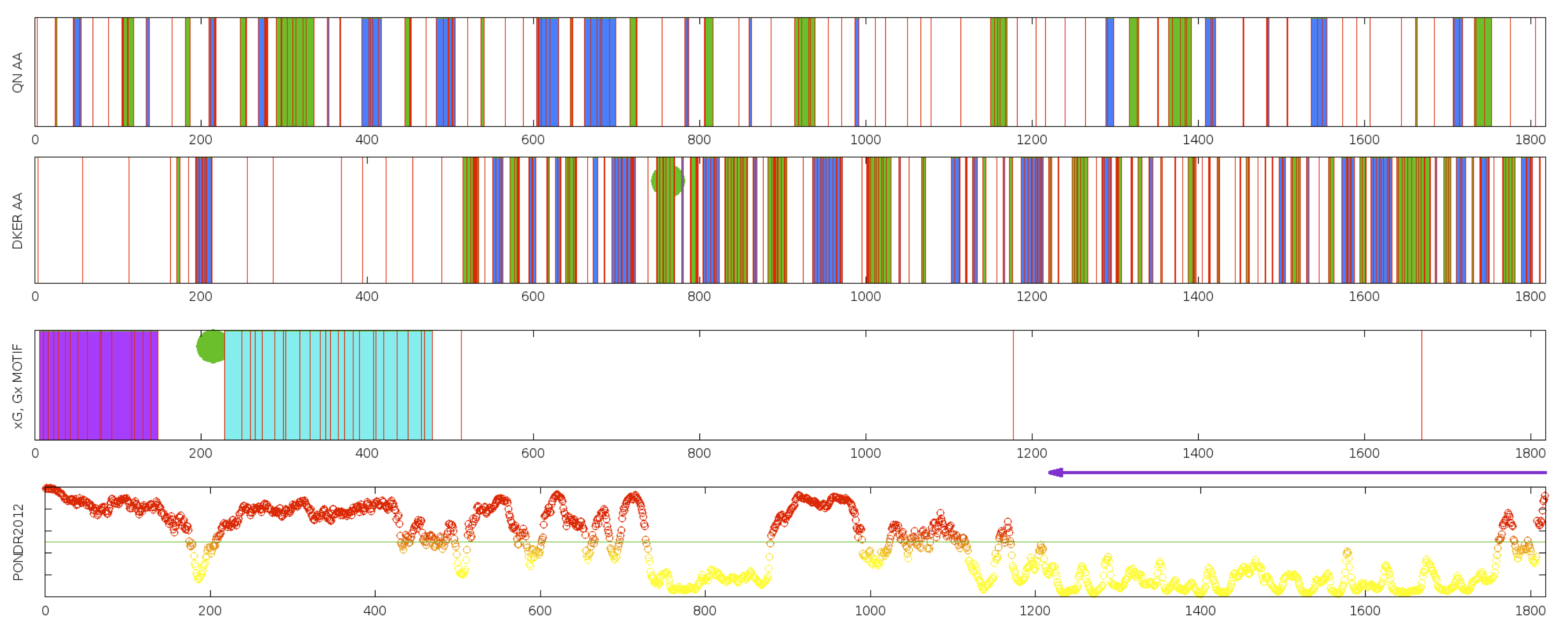

Supplement: Figure S16 — FG nucleoporin from Homo sapiens, Nup98, from the green group. Amino acid sequence number is shown along the x-axis for all sub-charts. The first chart starting from the top shows QN amino acids as red vertical lines and their clusters colored alternately blue and green as a control. Similarly colored is the second chart which shows charged amino acids, while the third chart has FG motifs represented as red vertical lines with clusters represented by alternating purple and cyan regions. The fourth chart displays the propensity for protein disorder for a given AA as predicted by PONDR, with red representing high propensity and yellow representing low propensity. Green circles represent centers of masses of cluster regions and the purple arrow indicates disordered region to folded region polarity. (TIFF) [file pone.0073831.s016.tiff]

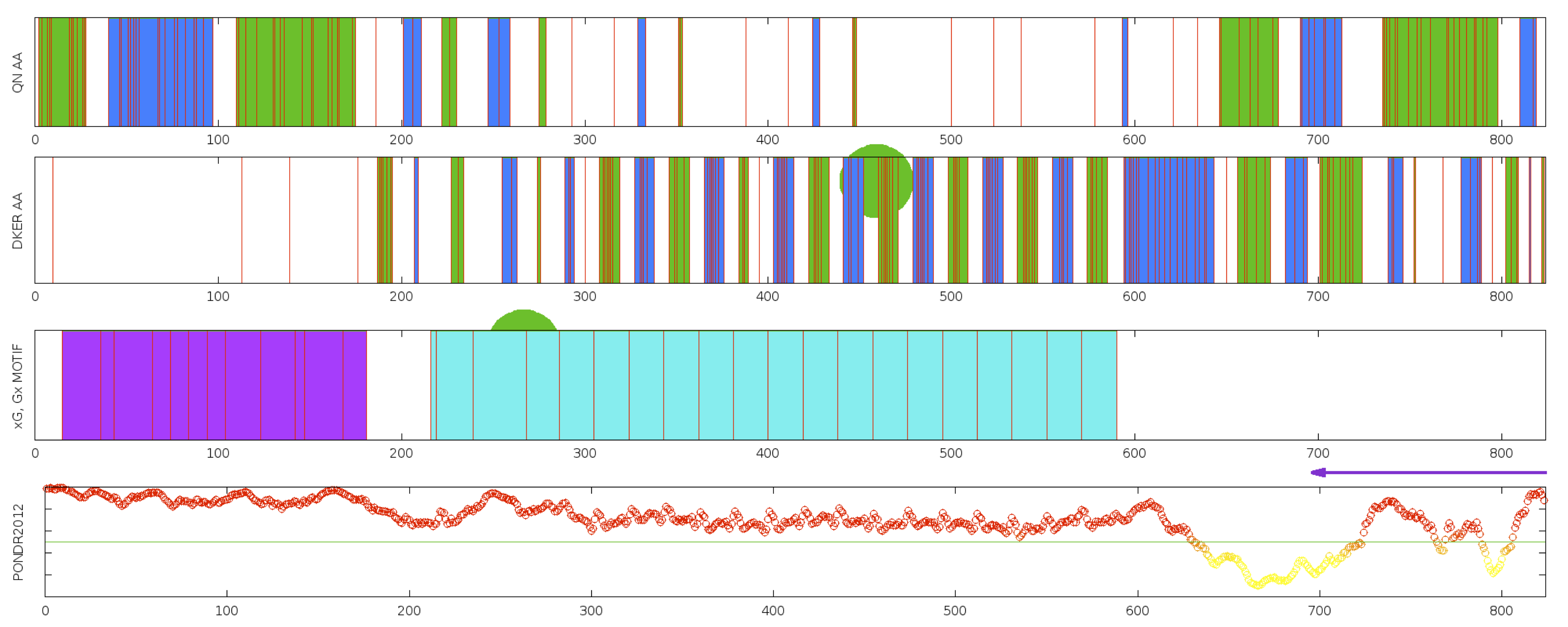

Supplement: Figure S17 — FG nucleoporin from S. cerevisiae, Nsp1, from the red group. Amino acid sequence number is shown along the x-axis for all sub-charts. The first chart starting from the top shows QN amino acids as red vertical lines and their clusters colored alternately blue and green as a control. Similarly colored is the second chart which shows charged amino acids, while the third chart has FG motifs represented as red vertical lines with clusters represented by alternating purple and cyan regions. The fourth chart displays the propensity for protein disorder for a given AA as predicted by PONDR, with red representing high propensity and yellow representing low propensity. Green circles represent centers of masses of cluster regions and the purple arrow indicates disordered region to folded region polarity. (TIFF) [file pone.0073831.s017.tiff]

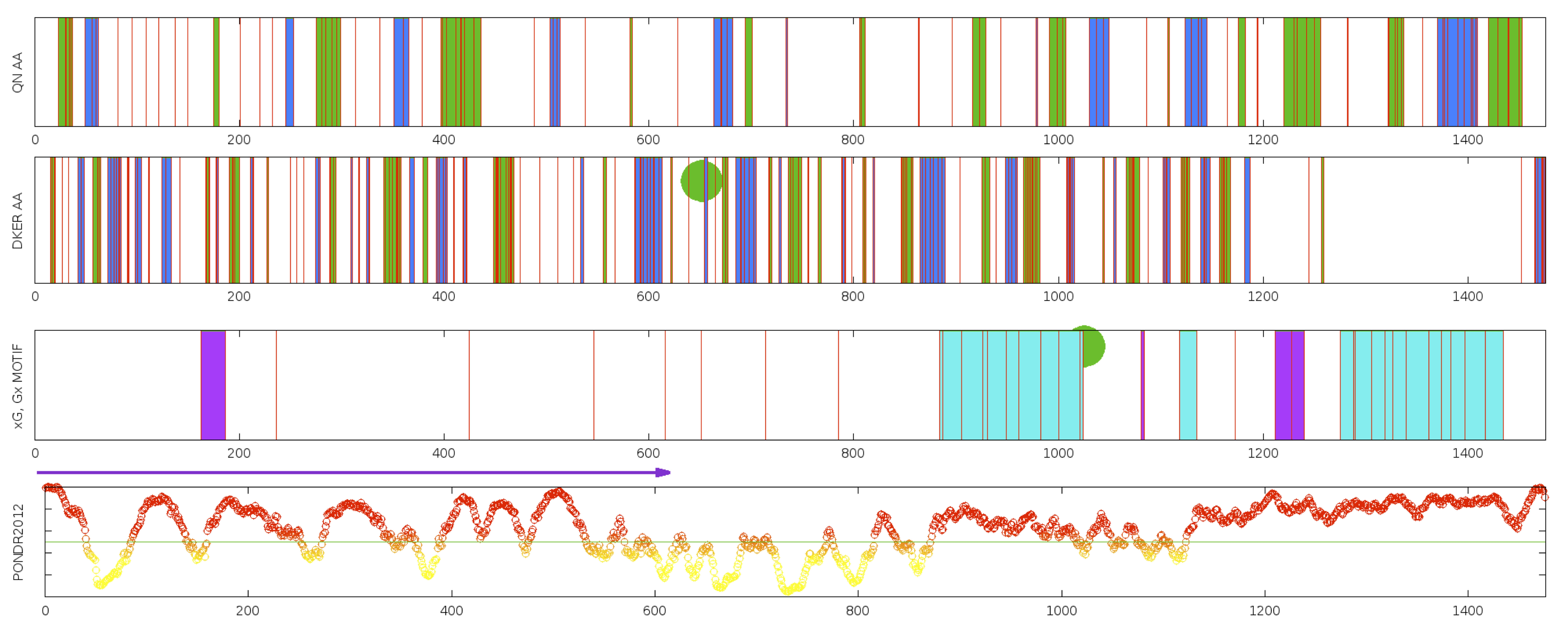

Supplement: Figure S18 — FG nucleoporin from Homo sapiens, Nup153, from the red group. Amino acid sequence number is shown along the x-axis for all sub-charts. The first chart starting from the top shows QN amino acids as red vertical lines and their clusters colored alternately blue and green as a control. Similarly colored is the second chart which shows charged amino acids, while the third chart has FG motifs represented as red vertical lines with clusters represented by alternating purple and cyan regions. The fourth chart displays the propensity for protein disorder for a given AA as predicted by PONDR, with red representing high propensity and yellow representing low propensity. Green circles represent centers of masses of cluster regions and the purple arrow indicates disordered region to folded region polarity. (TIFF) [file pone.0073831.s018.tiff]
