# Supplementary material for: Physical Motif Clustering within Intrinsically Disordered Nucleoporin Sequences Reveals Universal Functional Features
Source: PLoS One. 2013 Sep 16;8(9):e73831. doi: 10.1371/journal.pone.0073831 (PMC3774778; doi:10.1371/journal.pone.0073831)
Supplement: Text S1 — Supporting text. (PDF) [file pone.0073831.s020.pdf]

SUPPLEMENTARY TEXT

This is the Supplementary Text for 'Physical motif clustering within intrinsically disordered nucleoporin sequences reveals universal functional features.'

Contents

1 Exploration of different biophysical motif clusterings for FG nups 1

2 Identification of FG nups 2

3 Disordered regions of FG nups which exclude FG clusters but contain charge clusters are neutral on average 6

4 Net charge of folded regions of FG nups 7

5 Clustering among individual FG nups 7

6 Overview of FG nups in each clustering group for Humans and S. cerevisiae with representative visualizations 13

7 List of the 252 FG nup species analyzed 18

8 Spatial Sequence Correlation of FG and GF motifs 19

1 Exploration of different biophysical motif clusterings for FG nups

A series of clustering possibilities was considered starting with the hydrophobic/small amino acids (LVIMCAGSTPFYW) versus the hydrophilic amino acids (EDNQKRH) defined in the two letter reduced amino acid alphabet derived by grouping and averaging the full similarity matrix elements of BLOSUM50 [1]. This group showed considerable overlap between clustering types. The next case considered was the hydrophobic/small amino acids versus charged amino acids (EDKR), which performed in a similar manner. Considered next was the F modulo class of F equivalent AAs in the 4 letter BLOSUM derived alphabet versus the charged amino acids. This case was considered given the high enrichment of F amino acids in FG nups, and for the tendency of FG nups to have high density charged amino acids disordered regions which strongly affects polymer behavior. Possibly the most physically relevant property of FG nups is their FG motifs whose biochemical function is to bind transport receptors. This property was then clustered against the charged amino acids, which resulted in the most disjoint clustering class of all of the clustering groups tested, with 70% of FG clusters found to be more than 90% disjoint from charge clusters and nearly no FG clusters having greater than 90% overlap with charge clusters (Fig. S1). The next biophysical properties considered were simplified versions of FG motif vs. charged amino acids which yielded lower values of disjointness, similar to the results obtained with the other generalizations of these motifs considered. These other cases involved further simplified motif clustering possibilities including reducing in the FG motif to simply F as well as representing charges only by the most highly enriched charged amino acid in nups, K [2].

Explorations across possible biophysical clustering types was limited to proteins from distantly related Saccharomyces species and Homo sapiens FG nup proteins contained within the Uniprot (www.uniprot.org) database to conserve computational resources, while a full exploration of all nucleoporins was done only for FG and charged AAs. The 85 Saccharomyces and Homo genus proteins analyzed are listed in SI Data.

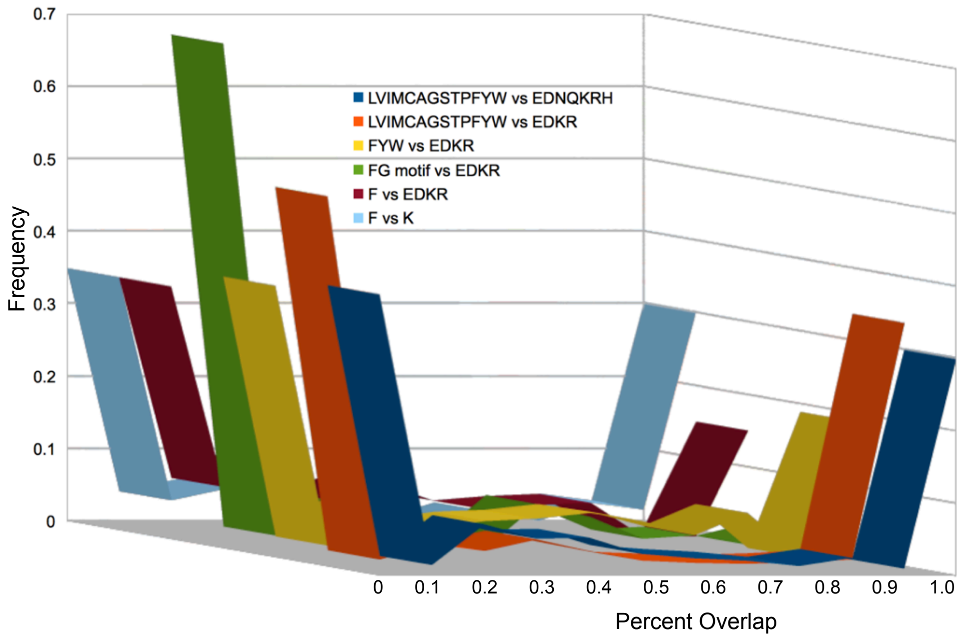

Supplementary Discussion Fig. 1: Histogram of percentage overlap for generalized and simplified FG motifs (LVIMCAGSTPFYW, FYW, and F) versus various characterizations of polar amino acids (EDNQKRH, EDKR, and K). Analyzing FG nups using FG motif versus charged AAs results in a clustering with the least degree of overlap, and is therefore the simplest and easiest to interpret.

2 Identification of FG nups

Initially all NUP proteins were analyzed for percent disorder and total FG density which resulted in a relatively continuous distribution as can be seen in Fig. S2.

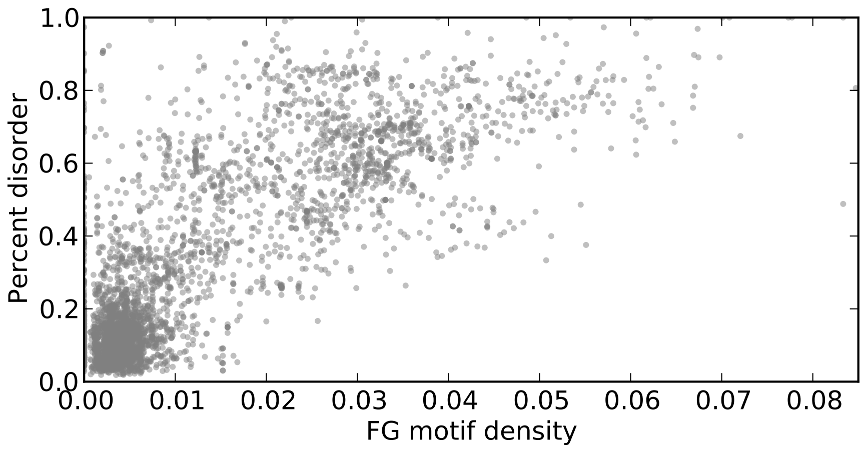

Supplementary Discussion Fig. 2: Continuous distribution of nups across NUP in terms of percent disorder (y-axis) and FG density in FG motifs/AA (x-axis).

In contrast, when the NUP proteins were restricted to those with 400 or more AAs and with fragment proteins removed, two distinct groups of nucleoporins were found to arise naturally, a group with low percentage disorder and low FG density, and another group with relatively high percentage disorder and relatively high FG density. Inspection of the names of the proteins showed that the low percentage disorder and low FG density group consists of karyopherin and structural nups, while the other group conyained known FG nups. An exhaustive and systematic labeling of all nucleoporins for Saccharomyces Cerevisiae yeast and humans confirmed that known karyopherin and structural nups formed one group, while the other group consisted of known FG nups. We took proteins in the NUP group with greater than 0.15 FG/AA linear motif density and greater than 30% disorder to be the FG nups which we analyzed in this study.

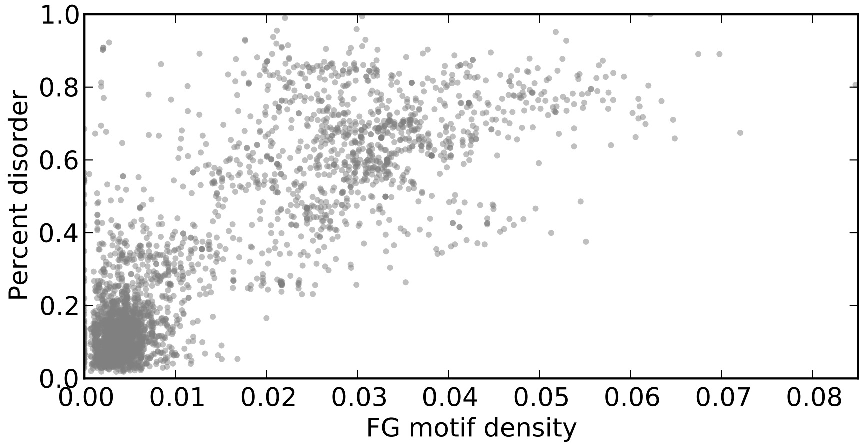

Supplementary Discussion Fig. 3: Natural split for nups for NUP restricted to proteins with greater than 400 AA at roughly greater than 10% FG/AA FG motif density and greater than 30% protein disorder.

Examining solely the Saccharomyces Cerevisiae nucleoporins confirms the split between FG nups and the structural/transport proteins as seen in Fig. S4.

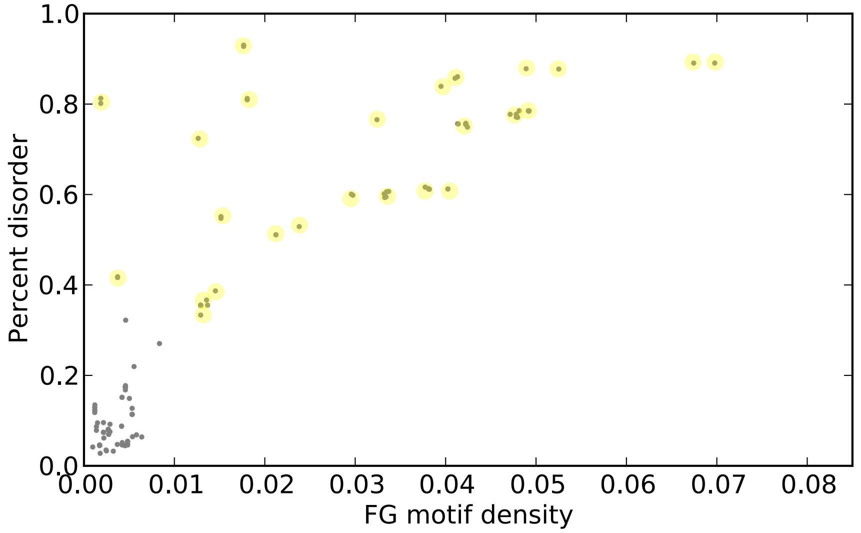

Supplementary Discussion Fig. 4: Natural split for Baker's Yeast, with 400 AA restriction. Yellow circles highlights refer to known FG nups while grey dots which are not highlighted represent known structural/transport proteins.

Examining solely the human nucleoporins confirms the split between FG nups and the structural/transport proteins as seen in Fig. S5.

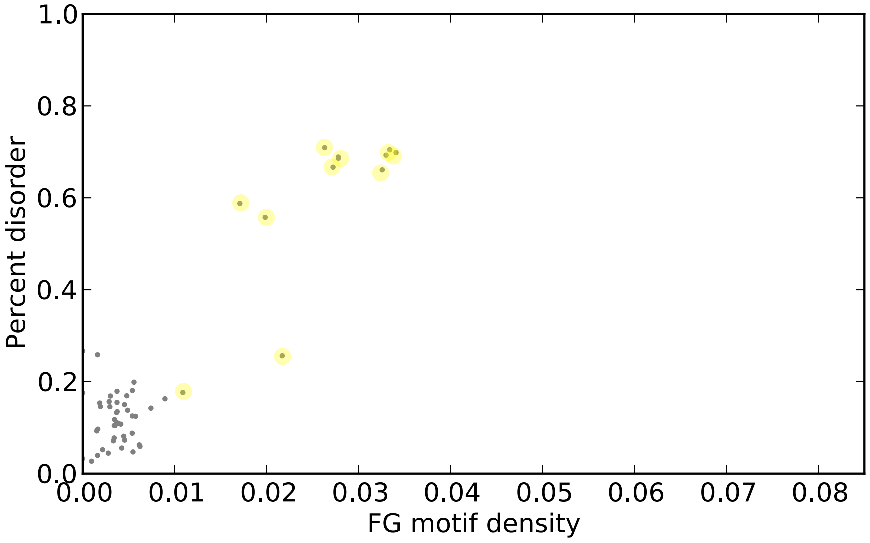

Supplementary Discussion Fig. 5: Natural split for humans, with 400 AA restriction. Yellow circles highlights refer to known FG nups while grey dots which are not highlighted represent known structural/transport proteins.

3 Disordered regions of FG nups which exclude FG clusters but contain charge clusters are neutral on average

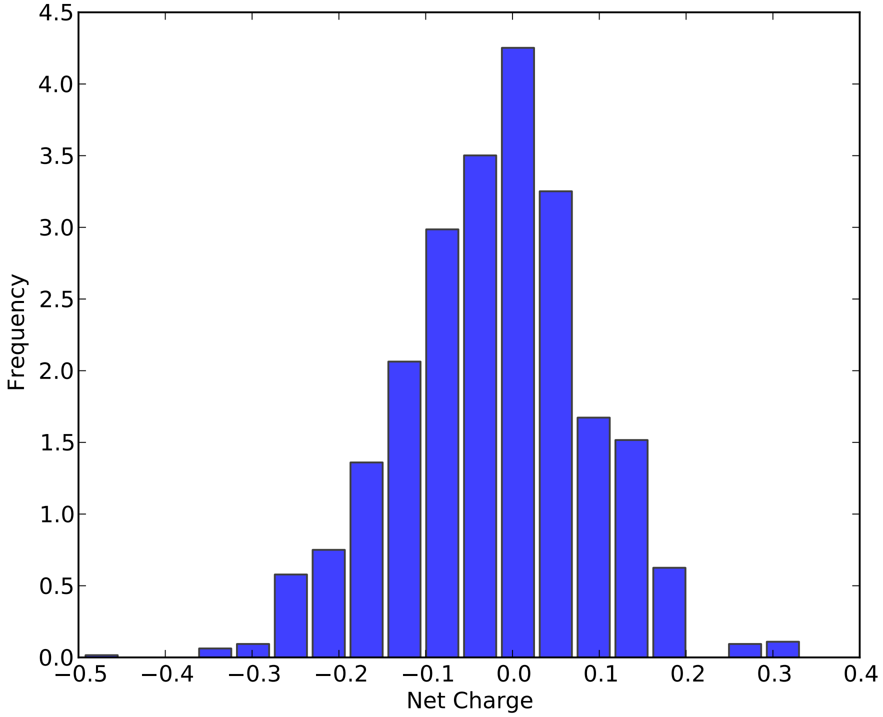

Supplementary Discussion Fig. 6: Net charge/AA for charged disordered charged regions of FG nups. The net charge for disordered charged regions of FG nups histogram is symmetric around zero net charge indicating that on average these regions are not selected for any net charge and on average are charge neutral.

4 Net charge of folded regions of FG nups

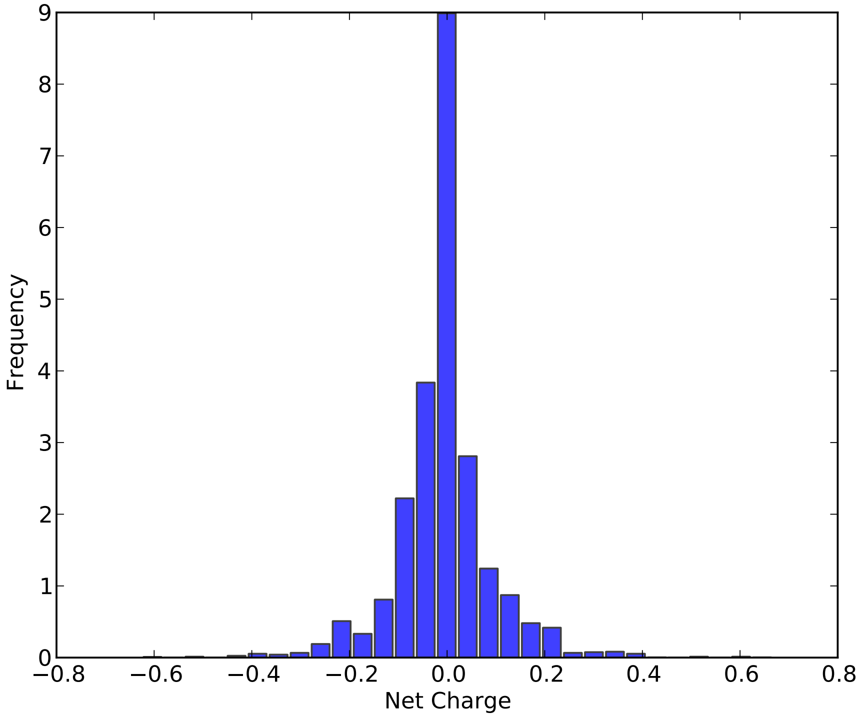

Supplementary Discussion Fig. 7: Net charge/AA for folded regions of FG nups is symmetric around zero net charge indicating that on average these regions are not selected for any net charge and on average are neutrally charged.

5 Clustering among individual FG nups

The 1,167 FG nups analyzed were clustered using the PreDeCon algorithm [3] over a five dimensional space consisting of FG-Charge cluster overlap in FG nups, FG-Charge cluster polarity, Folded-Disordered region polarity, percent of disordered region composed of charged AA clusters, and topological complexity. Setting the minimum cluster size to 50 proteins allowed for a large scale overview of how the 1167 FG nups organize themselves in this five dimensional space, which resulted in four major groupings of FG nups.

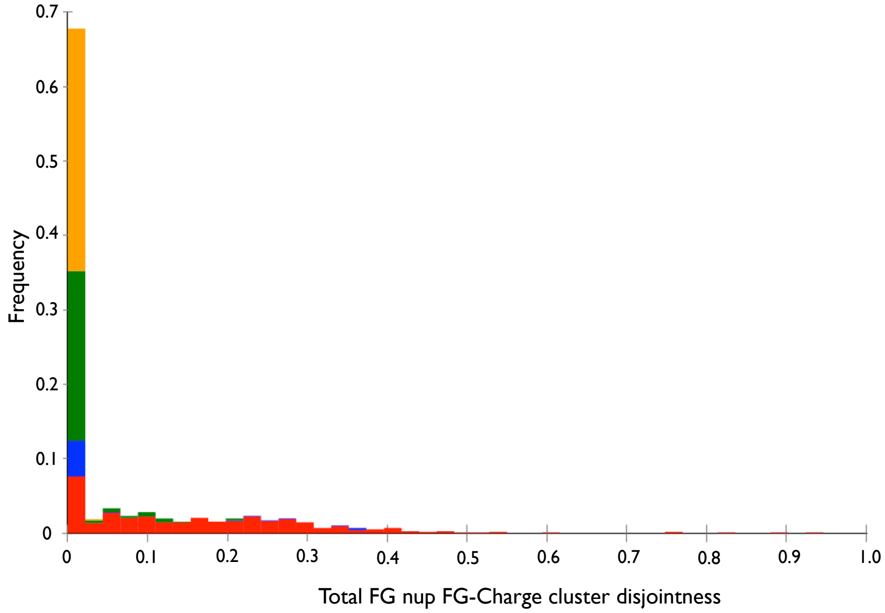

Supplementary Discussion Fig. 8: Histogram of FG-charge cluster percentage overlap in FG nups. Density clustering separates FG nups into 4 distinct groups, which are labeled, yellow, green, red and blue. Along the disjointness axis (x-axis) the red, green, and blue cluster groups aggregate at very high levels of disjointness, while the red group tends to have moderate levels of overlap.

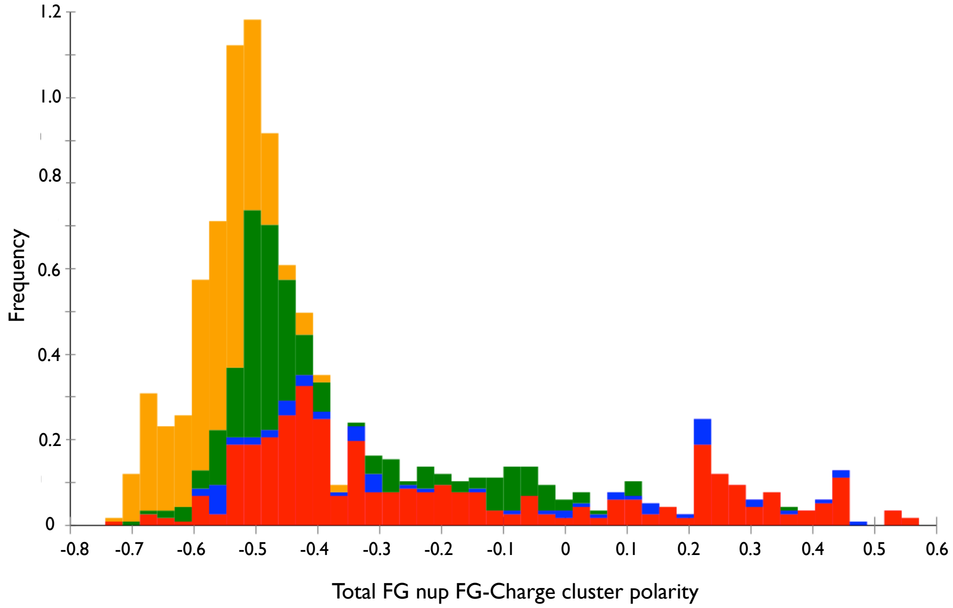

Supplementary Discussion Fig. 9: Histogram of FG-Charge cluster polarity. Density clustering separates FG nups into 4 distinct groups, which are shown in yellow, green, red and blue. Along the FG-Charge polarity axis positive polarity nups are nearly entirely from the red group, while other groups have significant negative polarity.

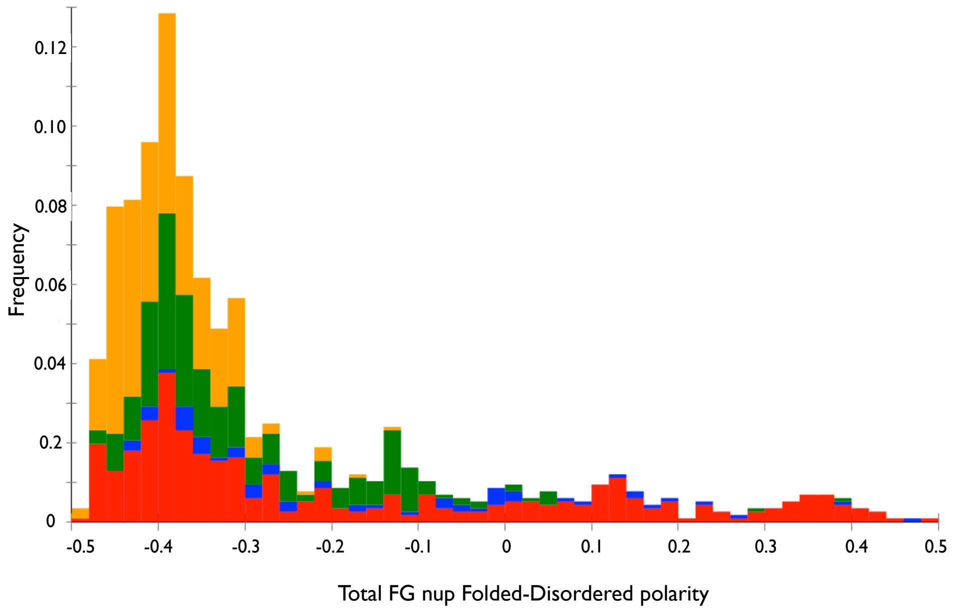

Supplementary Discussion Fig. 10: Histogram of FG nup Folded-Disordered region polarity. Density clustering separates FG nups into 4 distinct groups, which are labeled, yellow, green, red and blue. The positive polarity nups are nearly entirely from the red group, while other groups have significant negative polarity.

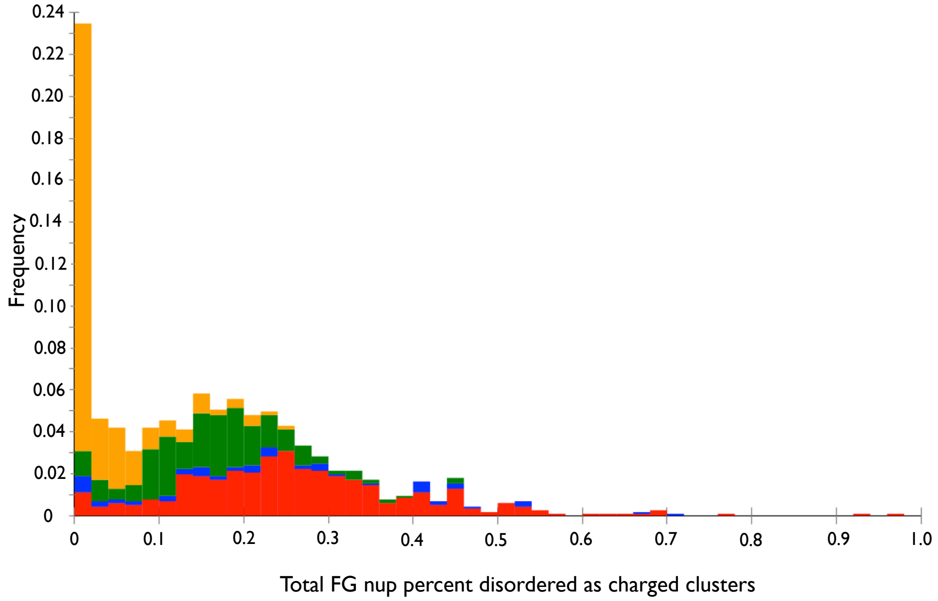

Supplementary Discussion Fig. 11: Histogram of the percent of disordered region composed of charged AA clusters for FG nups. Density clustering separates FG nups into 4 distinct groups, shown in yellow, green, red and blue. Along the percentage charged cluster axis the yellow group few charged amino acid clusters in the disordered regions, while the other three groups have significant charged disordered regions.

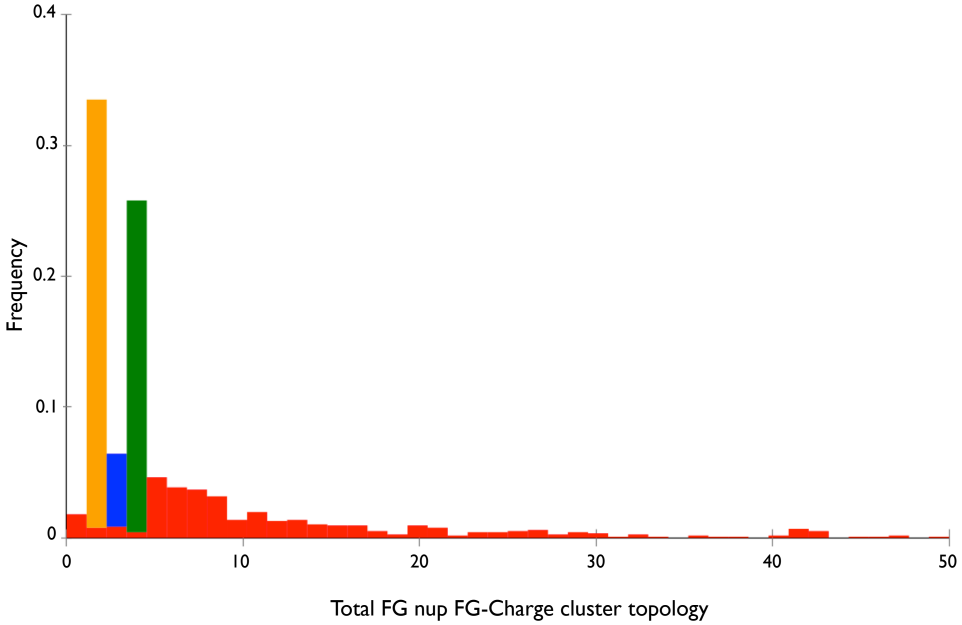

Supplementary Discussion Fig. 12: Histogram of the topological complexity of FG nups. Density clustering separates FG nups into 4 distinct groups, shown in yellow, green, red and blue. Along the topological complexity axis the yellow group has exclusively a topological complexity of 1, the blue group a topological complexity of 2, the green group a topological complexity of 3, while the red group has a topological complexity distributed over a range of higher values.

6 Overview of FG nups in each clustering group for Humans and S. cerevisiae with representative visualizations

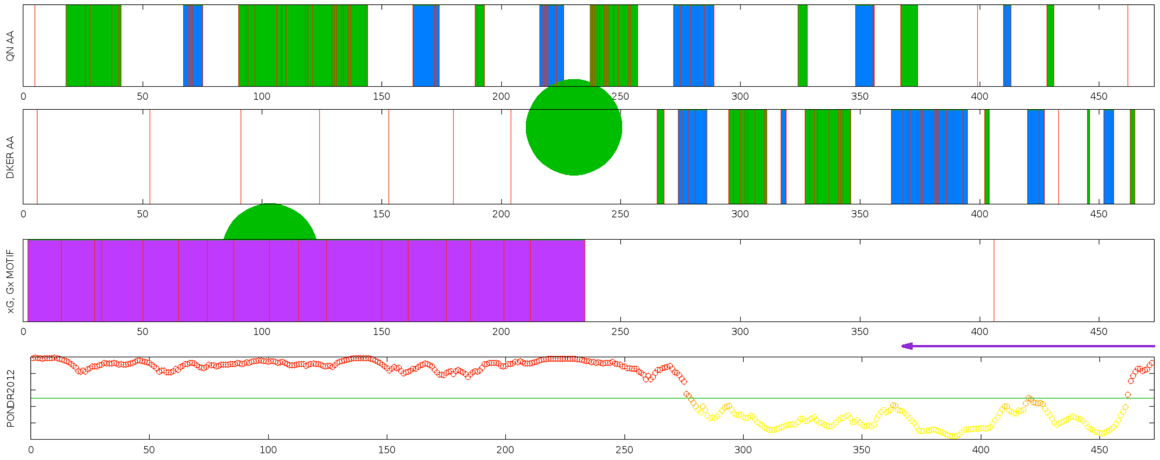

Supplementary Discussion Fig. 13: FG nucleoporin from S. cerevisiae, Nup49, from the yellow group. Amino acid sequence number is shown along the x-axis for all sub-charts. The first chart starting from the top shows QN amino acids as red vertical lines and their clusters colored alternately blue and green as a control. Similarly colored is the second chart which shows charged amino acids, while the third chart has FG motifs represented as red vertical lines with clusters represented by alternating purple and cyan regions. The fourth chart displays the propensity for protein disorder for a given AA as predicted by PONDR, with red representing high propensity and yellow representing low propensity. Green circles represent centers of masses of cluster regions and the purple arrow indicates disordered region to folded region polarity.

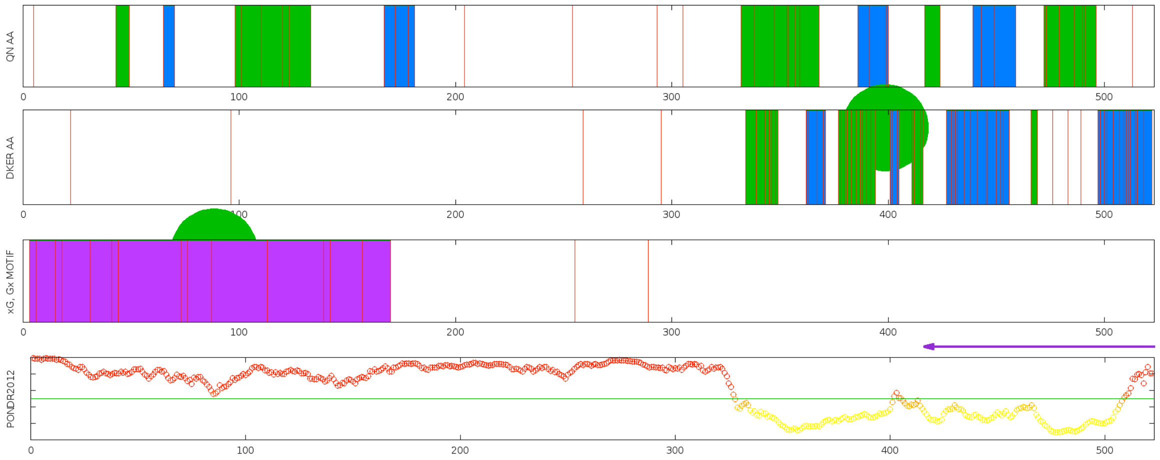

Supplementary Discussion Fig. 14: FG nucleoporin from Homo sapiens, Nup62, from the yellow group. Amino acid sequence number is shown along the x-axis for all sub-charts. The first chart starting from the top shows QN amino acids as red vertical lines and their clusters colored alternately blue and green as a control. Similarly colored is the second chart which shows charged amino acids, while the third chart has FG motifs represented as red vertical lines with clusters represented by alternating purple and cyan regions. The fourth chart displays the propensity for protein disorder for a given AA as predicted by PONDR, with red representing high propensity and yellow representing low propensity. Green circles represent centers of masses of cluster regions and the purple arrow indicates disordered region to folded region polarity.

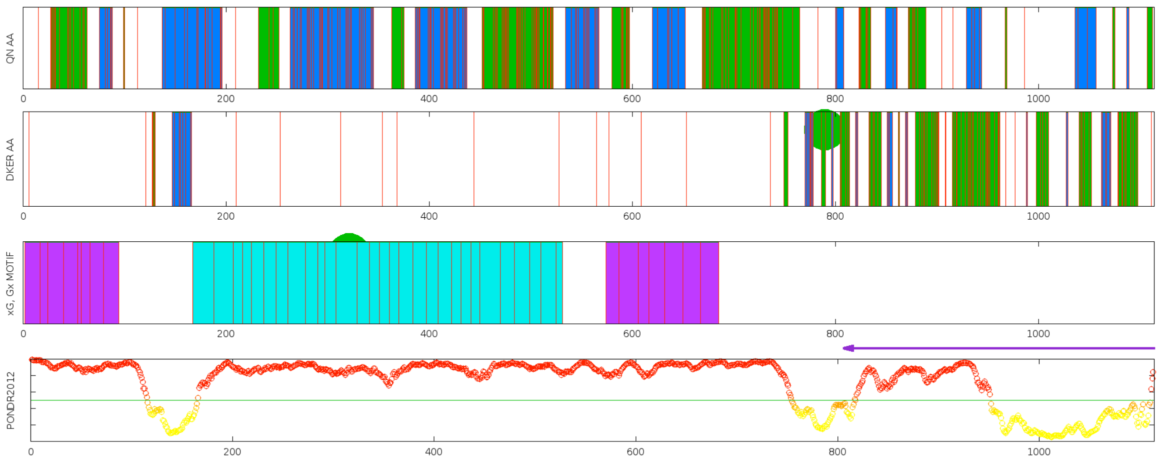

Supplementary Discussion Fig. 15: FG nucleoporin from S. cerevisiae, Nup116, from the green group. Amino acid sequence number is shown along the x-axis for all sub-charts. The first chart starting from the top shows QN amino acids as red vertical lines and their clusters colored alternately blue and green as a control. Similarly colored is the second chart which shows charged amino acids, while the third chart has FG motifs represented as red vertical lines with clusters represented by alternating purple and cyan regions. The fourth chart displays the propensity for protein disorder for a given AA as predicted by PONDR, with red representing high propensity and yellow representing low propensity. Green circles represent centers of masses of cluster regions and the purple arrow indicates disordered region to folded region polarity.

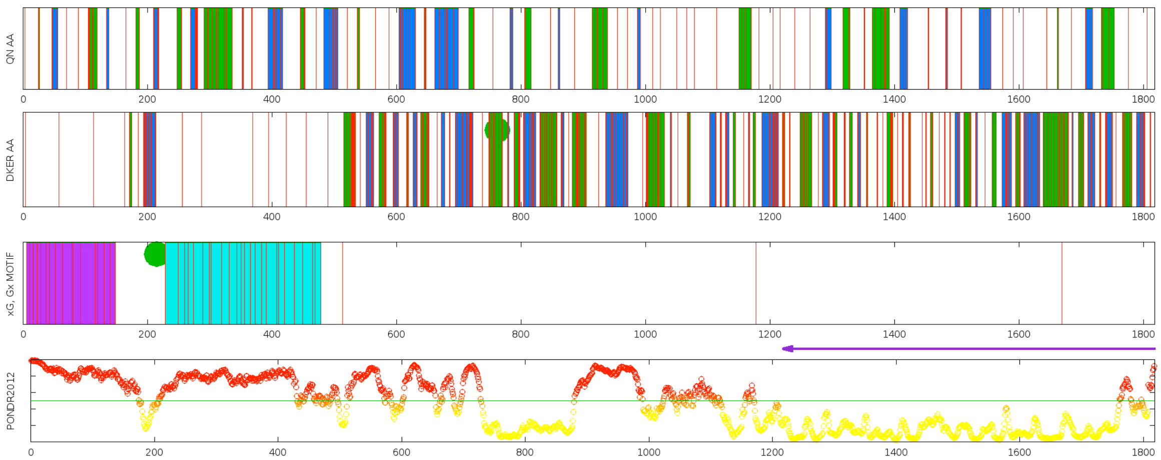

Supplementary Discussion Fig. 16: FG nucleoporin from Homo sapiens, Nup98, from the green group. Amino acid sequence number is shown along the x-axis for all sub-charts. The first chart starting from the top shows QN amino acids as red vertical lines and their clusters colored alternately blue and green as a control. Similarly colored is the second chart which shows charged amino acids, while the third chart has FG motifs represented as red vertical lines with clusters represented by alternating purple and cyan regions. The fourth chart displays the propensity for protein disorder for a given AA as predicted by PONDR, with red representing high propensity and yellow representing low propensity. Green circles represent centers of masses of cluster regions and the purple arrow indicates disordered region to folded region polarity.

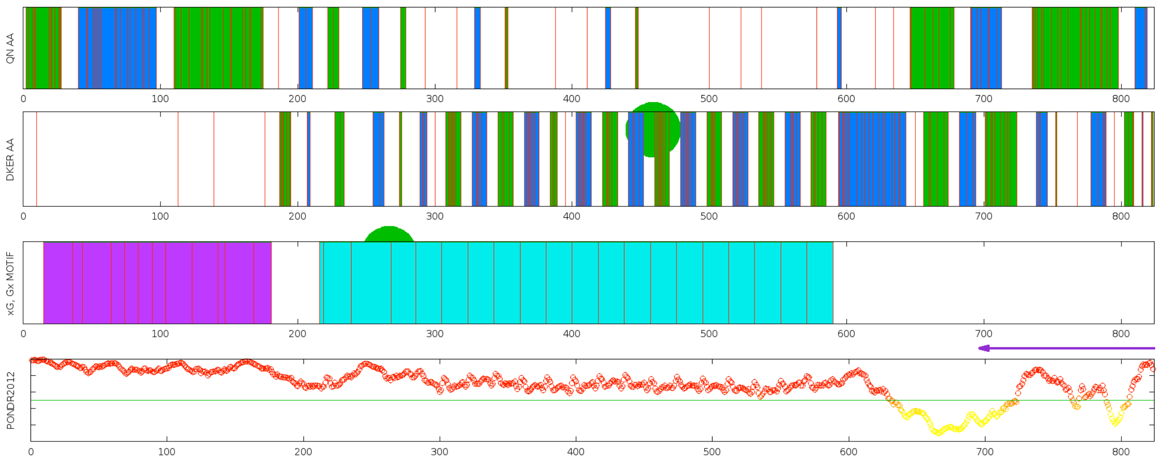

Supplementary Discussion Fig. 17: FG nucleoporin from S. cerevisiae, Nsp1, from the red group. Amino acid sequence number is shown along the x-axis for all sub-charts. The first chart starting from the top shows QN amino acids as red vertical lines and their clusters colored alternately blue and green as a control. Similarly colored is the second chart which shows charged amino acids, while the third chart has FG motifs represented as red vertical lines with clusters represented by alternating purple and cyan regions. The fourth chart displays the propensity for protein disorder for a given AA as predicted by PONDR, with red representing high propensity and yellow representing low propensity. Green circles represent centers of masses of cluster regions and the purple arrow indicates disordered region to folded region polarity.

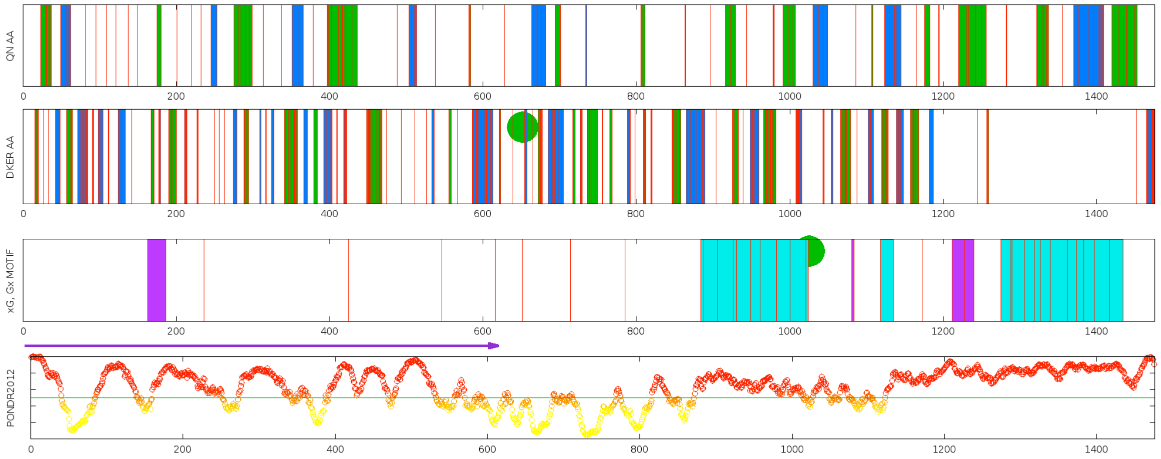

Supplementary Discussion Fig. 18: FG nucleoporin from Homo sapiens, Nup153, from the red group. Amino acid sequence number is shown along the x-axis for all sub-charts. The first chart starting from the top shows QN amino acids as red vertical lines and their clusters colored alternately blue and green as a control. Similarly colored is the second chart which shows charged amino acids, while the third chart has FG motifs represented as red vertical lines with clusters represented by alternating purple and cyan regions. The fourth chart displays the propensity for protein disorder for a given AA as predicted by PONDR, with red representing high propensity and yellow representing low propensity. Green circles represent centers of masses of cluster regions and the purple arrow indicates disordered region to folded region polarity.

Following is a list of well known FG nups from Humans and S. cerevisiae and their corresponding FG nup cluster. The full list of all FG nups analyzed and their cluster group is in SI Data.

List of proteins in the Red group:

tr|B3KUL1|B3KUL1 HUMAN cDNA FLJ40132 fis, clone TESTI2012155, highly similar to NUCLEOPORIN-LIKE PROTEIN RIP OS=Homo sapiens PE=2 SV=1

tr|B4DIK2|B4DIK2 HUMAN cDNA FLJ60565, highly similar to Nuclear pore complex protein Nup153 OS=Homo sapiens PE=2 SV=1

tr|B4DHC5|B4DHC5 HUMAN cDNA FLJ57927, highly similar to Nucleoporin-like protein RIP OS=Homo sapiens PE=2 SV=1

sp|P35658|NU214 HUMAN Nuclear pore complex protein Nup214 OS=Homo sapiens GN=NUP214 PE=1 SV=2

sp|P49790|NU153 HUMAN Nuclear pore complex protein Nup153 OS=Homo sapiens GN=NUP153 PE=1 SV=2

sp|P49792|RBP2 HUMAN E3 SUMO-protein ligase RanBP2 OS=Homo sapiens GN=RANBP2 PE=1 SV=2

sp|P52594|AGFG1 HUMAN Arf-GAP domain and FG repeat-containing protein 1 OS=Homo sapiens GN=AGFG1 PE=1 SV=2

sp|Q9UKX7|NUP50 HUMAN Nuclear pore complex protein Nup50 OS=Homo sapiens GN=NUP50 PE=1 SV=2

sp|Q96HA1|P121A HUMAN Nuclear envelope pore membrane protein POM 121 OS=Homo sapiens GN=POM121 PE=1 SV=2

tr|Q7Z743|Q7Z743 HUMAN Nucleoporin 153kDa OS=Homo sapiens GN=NUP153 PE=2 SV=1

tr|F6QR24|F6QR24 HUMAN Nuclear pore complex protein Nup153 OS=Homo sapiens GN=NUP153 PE=4 SV=1

tr|A6ZU88|A6ZU88 YEAS7 Nuclear pore complex subunit OS=Saccharomyces cerevisiae (strain YJM789) GN=NUP145 PE=4 SV=1

tr|A6ZPT6|A6ZPT6 YEAS7 Nuclear pore complex subunit OS=Saccharomyces cerevisiae (strain YJM789) GN=NSP1 PE=4 SV=1

tr|A7A1L6|A7A1L6 YEAS7 Nucleoporin OS=Saccharomyces cerevisiae (strain YJM789) GN=NUP2 PE=4 SV=1

tr|A6ZZP8|A6ZZP8 YEAS7 Nuclear pore complex subunit OS=Saccharomyces cerevisiae (strain YJM789) GN=NUP100 PE=4 SV=1

tr|A6ZVG2|A6ZVG2 YEAS7 Nucleoporin OS=Saccharomyces cerevisiae (strain YJM789) GN=NUP159 PE=4 SV=1

tr|B3LJE4|B3LJE4 YEAS1 Nucleoporin NUP1 OS=Saccharomyces cerevisiae (strain RM11-1a) GN=SCRG 01496 PE=4 SV=1

tr|B3LG91|B3LG91 YEAS1 Nucleoporin NUP42 OS=Saccharomyces cerevisiae (strain RM11-1a) GN=SCRG 00328 PE=4 SV=1

tr|B3LGY3|B3LGY3 YEAS1 Nucleoporin ASM4 OS=Saccharomyces cerevisiae (strain RM11-1a) GN=SCRG 00585 PE=4 SV=1

tr|B3LHF7|B3LHF7 YEAS1 Nucleoporin NUP145 OS=Saccharomyces cerevisiae (strain RM11-1a) GN=SCRG 01090 PE=4 SV=1

tr|B3RHK8|B3RHK8 YEAS1 Nucleoporin OS=Saccharomyces cerevisiae (strain RM11-1a) GN=SCRG 04280 PE=4 SV=1

tr|B3LTW2|B3LTW2 YEAS1 Nucleoporin OS=Saccharomyces cerevisiae (strain RM11-1a) GN=SCRG 05287 PE=4 SV=1

tr|B3LR23|B3LR23 YEAS1 Nucleoporin NUP100/NSP100 OS=Saccharomyces cerevisiae (strain RM11-1a) GN=SCRG 03952 PE=4 SV=1

tr|Q6FPU8|Q6FPU8 CANGA Similar to uniprot|P14907 Saccharomyces cerevisiae YJL041w NSP1 OS=Candida glabrata (strain ATCC 2001 / CBS 138 / JCM 3761 / NBRC 0622 / NRRL Y-65) GN=CAGL0J00781g PE=4 SV=1

sp|P14907|NSP1 YEAST Nucleoporin NSP1 OS=Saccharomyces cerevisiae (strain ATCC 204508 / S288c) GN=NSP1 PE=1 SV=1

sp|P20676|NUP1 YEAST Nucleoporin NUP1 OS=Saccharomyces cerevisiae (strain ATCC 204508 / S288c) GN=NUP1 PE=1 SV=1

sp|P32499|NUP2 YEAST Nucleoporin NUP2 OS=Saccharomyces cerevisiae (strain ATCC 204508 / S288c) GN=NUP2 PE=1 SV=2

sp|P40477|NU159 YEAST Nucleoporin NUP159 OS=Saccharomyces cerevisiae (strain ATCC 204508 / S288c) GN=NUP159 PE=1 SV=1

sp|P49686|NUP42 YEAST Nucleoporin NUP42 OS=Saccharomyces cerevisiae (strain ATCC 204508 / S288c) GN=NUP42 PE=1 SV=1

sp|P49687|NU145 YEAST Nucleoporin NUP145 OS=Saccharomyces cerevisiae (strain ATCC 204508 / S288c) GN=NUP145 PE=1 SV=1

sp|Q05166|NUP59 YEAST Nucleoporin ASM4 OS=Saccharomyces cerevisiae (strain ATCC 204508 / S288c) GN=ASM4 PE=1 SV=1

sp|Q03790|NUP53 YEAST Nucleoporin NUP53 OS=Saccharomyces cerevisiae (strain ATCC 204508 / S288c) GN=NUP53 PE=1 SV=1

tr|Q6FPU8|Q6FPU8 CANGA Similar to uniprot|P14907 Saccharomyces cerevisiae YJL041w NSP1 OS=Candida glabrata (strain ATCC 2001 / CBS 138 / JCM 3761 / NBRC 0622 / NRRL Y-65) GN=CAGL0J00781g PE=4 SV=1

tr|C7GW26|C7GW26 YEAS2 Nup145p OS=Saccharomyces cerevisiae (strain JAY291) GN=NUP145 PE=4 SV=1

tr|C8ZBH3|C8ZBH3 YEAS8 Nsp1p OS=Saccharomyces cerevisiae (strain Lalvin EC1118 / Prise de mousse) GN=EC1118 1J11 2157g PE=4 SV=2

tr|C8Z8F6|C8Z8F6 YEAS8 Nup145p OS=Saccharomyces cerevisiae (strain Lalvin EC1118 / Prise de mousse) GN=EC1118 1G1 1959g PE=4 SV=1

tr|E7Q648|E7Q648 YEASB Nup100p OS=Saccharomyces cerevisiae (strain FostersB) GN=FOSTERSB 2851 PE=4 SV=1

tr|E7KNE8|E7KNE8 YEASL Nup145p OS=Saccharomyces cerevisiae (strain Lalvin QA23) GN=QA23 1700 PE=4 SV=1

tr|E7NJQ4|E7NJQ4 YEASO Nsp1p OS=Saccharomyces cerevisiae (strain FostersO) GN=FOSTERSO 2551 PE=4 SV=1

tr|E7KEX2|E7KEX2 YEASA Nup100p OS=Saccharomyces cerevisiae (strain AWRI796) GN=AWRI796 2893 PE=4 SV=1

tr|G2WGX7|G2WGX7 YEASK K7 Nsp1p OS=Saccharomyces cerevisiae (strain Kyokai no. 7 / NBRC 101557) GN=K7 NSP1 PE=4 SV=1

tr|G2WE08|G2WE08 YEASK K7 Nup145p OS=Saccharomyces cerevisiae (strain Kyokai no. 7 / NBRC 101557) GN=K7 NUP145 PE=4 SV=1

tr|H0GG71|H0GG71 9SACH Nup145p OS=Saccharomyces cerevisiae x Saccharomyces kudriavzevii VIN7 GN=VIN7 1730 PE=4 SV=1

tr|H0GJ66|H0GJ66 9SACH Nup100p OS=Saccharomyces cerevisiae x Saccharomyces kudriavzevii VIN7 GN=VIN7 2942 PE=4 SV=1

List of proteins in the Blue group:

sp|O15504|NUPL2 HUMAN Nucleoporin-like protein 2 OS=Homo sapiens GN=NUPL2 PE=1 SV=1

tr|Q3B7J4|Q3B7J4 HUMAN Nucleoporin like 2 OS=Homo sapiens GN=NUPL2 PE=2 SV=1

tr|B2R7I1|B2R7I1 HUMAN cDNA, FLJ93452, highly similar to Homo sapiens nucleoporin like 2 (NUPL2), mRNA OS=Homo sapiens PE=2 SV=1

tr|B4DWF8|B4DWF8 HUMAN cDNA FLJ53414, highly similar to Nuclear pore complex protein Nup98-Nup96precursor OS=Homo sapiens PE=2 SV=1

List of proteins in the Yellow group:

sp|P37198|NUP62 HUMAN Nuclear pore glycoprotein p62 OS=Homo sapiens GN=NUP62 PE=1 SV=3

tr|B4E0K0|B4E0K0 HUMAN cDNA FLJ52820, highly similar to Nucleoporin p58/p45 OS=Homo sapiens PE=2 SV=1

tr|C9JDH3|C9JDH3 HUMAN Nucleoporin p58/p45 OS=Homo sapiens GN=NUPL1 PE=4 SV=2

tr|E7Q443|E7Q443 YEASB Nup57p OS=Saccharomyces cerevisiae (strain FostersB) GN=FOSTERSB 1849 PE=4 SV=1

sp|P48837|NUP57 YEAST Nucleoporin NUP57 OS=Saccharomyces cerevisiae (strain ATCC 204508 / S288c) GN=NUP57 PE=1 SV=1

tr|A6ZUD2|A6ZUD2 YEAS7 Nucleoporin OS=Saccharomyces cerevisiae (strain YJM789) GN=NUP57 PE=4 SV=1

tr|B3LIB0|B3LIB0 YEAS1 Nucleoporin OS=Saccharomyces cerevisiae (strain RM11-1a) GN=SCRG 00900 PE=4 SV=1

tr|B5VJ65|B5VJ65 YEAS6 YGR119Cp-like protein OS=Saccharomyces cerevisiae (strain AWRI1631) GN=AWRI1631 73440 PE=4 SV=1

tr|G2WEK1|G2WEK1 YEASK K7 Nup57p OS=Saccharomyces cerevisiae (strain Kyokai no. 7 / NBRC 101557) GN=K7 NUP57 PE=4 SV=1

tr|C7GUH3|C7GUH3 YEAS2 Nup57p OS=Saccharomyces cerevisiae (strain JAY291) GN=NUP57 PE=4 SV=1

tr|E7KCJ0|E7KCJ0 YEASA Nup57p OS=Saccharomyces cerevisiae (strain AWRI796) GN=AWRI796 1881 PE=4 SV=1

tr|C8Z910|C8Z910 YEAS8 Nup57p OS=Saccharomyces cerevisiae (strain Lalvin EC1118 / Prise de mousse) GN=EC1118 1G1 4335g PE=4 SV=1

tr|H0GGN3|H0GGN3 9SACH Nup57p OS=Saccharomyces cerevisiae x Saccharomyces kudriavzevii VIN7 GN=VIN7 1905 PE=4 SV=1

tr|E7NHZ5|E7NHZ5 YEASO Nup57p OS=Saccharomyces cerevisiae (strain FostersO) GN=FOSTERSO 1834 PE=4 SV=1

tr|H0GUL6|H0GUL6 9SACH Nup49p OS=Saccharomyces cerevisiae x Saccharomyces kudriavzevii VIN7 GN=VIN7 7019 PE=4 SV=1

tr|E7KCC2|E7KCC2 YEASA Nup49p OS=Saccharomyces cerevisiae (strain AWRI796) GN=AWRI796 1636 PE=4 SV=1

tr|E7QEJ1|E7QEJ1 YEASZ Nup49p OS=Saccharomyces cerevisiae (strain Zymaflore VL3) GN=VL3 1625 PE=4 SV=1

tr|A6ZU14|A6ZU14 YEAS7 Nuclear pore complex subunit OS=Saccharomyces cerevisiae (strain YJM789) GN=NUP49 PE=4 SV=1

tr|B3LHM4|B3LHM4 YEAS1 Nucleoporin NUP49/NSP49 OS=Saccharomyces cerevisiae (strain RM11-1a) GN=SCRG 01163 PE=4 SV=1

tr|C7GPN5|C7GPN5 YEAS2 Nup49p OS=Saccharomyces cerevisiae (strain JAY291) GN=NUP49 PE=4 SV=1

tr|C8Z879|C8Z879 YEAS8 Nup49p OS=Saccharomyces cerevisiae (strain Lalvin EC1118 / Prise de mousse) GN=EC1118 1G1 1068g PE=4 SV=1

tr|E7KN89|E7KN89 YEASL Nup49p OS=Saccharomyces cerevisiae (strain Lalvin QA23) GN=QA23 1628 PE=4 SV=1

tr|E7LUN9|E7LUN9 YEASV Nup49p OS=Saccharomyces cerevisiae (strain VIN 13) GN=VIN13 1619 PE=4 SV=1

tr|H0GG42|H0GG42 9SACH Nup49p OS=Saccharomyces cerevisiae x Saccharomyces kudriavzevii VIN7 GN=VIN7 1656 PE=4 SV=1

sp|Q02199|NUP49 YEAST Nucleoporin NUP49/NSP49 OS=Saccharomyces cerevisiae (strain ATCC 204508 / S288c) GN=NUP49 PE=1 SV=1

tr|G2WDT3|G2WDT3 YEASK K7 Nup49p OS=Saccharomyces cerevisiae (strain Kyokai no. 7 / NBRC 101557) GN=K7 NUP49 PE=4 SV=1

tr|B5VM90|B5VM90 YEAS6 YKL068Wp-like protein OS=Saccharomyces cerevisiae (strain AWRI1631) GN=AWRI1631 111600 PE=4 SV=1

tr|B5VPH5|B5VPH5 YEAS6 YMR047Cp-like protein OS=Saccharomyces cerevisiae (strain AWRI1631) GN=AWRI1631 131890 PE=4 SV=1

tr|G2WHW4|G2WHW4 YEASK K7 Nup100p OS=Saccharomyces cerevisiae (strain Kyokai no. 7 / NBRC 101557) GN=K7 NUP100 PE=4 SV=1

7 List of the 252 FG nup species analyzed

Acromyrmex echinatior, Aedes aegypti, Ailuropoda melanoleuca, Ajellomyces capsulata, Ajellomyces dermatitidis, Albugo laibachii, Amblyomma maculatum, Anguilla japonica, Anopheles darlingi, Anopheles gambiae, Arabidopsis lyrata subsp. lyrata, Arabidopsis thaliana, Arthrobotrys oligospora, Arthroderma benhamiae, Arthroderma gypseum, Arthroderma otae, Ashbya gossypii, Aspergillus clavatus, Aspergillus flavus, Aspergillus kawachii, Aspergillus niger, Aspergillus oryzae, Aspergillus terreus, Aureococcus anophagefferens, Babesia bovis, Batrachochytrium dendrobatidis, Blastocystis hominis, Bos taurus, Botryotinia fuckeliana, Branchiostoma floridae, Brugia malayi, Caenorhabditis brenneri, Caenorhabditis briggsae, Caenorhabditis elegans, Caenorhabditis japonica, Caenorhabditis remanei, Callithrix jacchus, Camponotus floridanus, Candida albicans, Candida dubliniensis, Candida glabrata, Candida parapsilosis, Candida tropicalis, Canis familiaris, Cavia porcellus, Chaetomium globosum, Chaetomium thermophilum, Chaetomium thermophilum var. thermophilum, Chlorella variabilis, Ciona intestinalis, Ciona savignyi, Clavispora lusitaniae, Clonorchis sinensis, Coccidioides posadasii, Colletotrichum graminicola, Colletotrichum higginsianum, Coprinopsis cinerea, Cordyceps militaris, Cricetulus griseus, Cryptococcus gattii serotype B, Cryptococcus neoformans var. neoformans serotype D, Cryptosporidium hominis, Cryptosporidium muris, Cryptosporidium parvum, Culex quinquefasciatus, Danaus plexippus, Danio rerio, Daphnia pulex, Daucus carota, Debaryomyces hansenii, Dicentrarchus labrax, Dictyostelium discoideum, Dictyostelium fasciculatum, Dictyostelium purpureum, Drosophila ananassae, Drosophila erecta, Drosophila grimshawi, Drosophila melanogaster, Drosophila mojavensis, Drosophila persimilis, Drosophila pseudoobscura pseudoobscura, Drosophila sechellia, Drosophila simulans, Drosophila virilis, Drosophila willistoni, Drosophila yakuba, Ectocarpus siliculosus, Emericella nidulans, Encephalitozoon cuniculi, Encephalitozoon intestinalis, Entamoeba dispar, Entamoeba histolytica, Equus caballus, Eremothecium cymbalariae, Exophiala dermatitidis, Fusarium oxysporum, Gallus gallus, Gasterosteus aculeatus, Giardia intestinalis, Glarea lozoyensis, Glossina morsitans morsitans, Gorilla gorilla gorilla, Grosmannia clavigera, Harpegnathos saltator, Heterocephalus glaber, Homo sapiens, Hordeum vulgare var. distichum, Hydra vulgaris, Hypocrea atroviridis, Hypocrea jecorina, Hypocrea virens, Ichthyophthirius multifiliis, Kazachstania africana, Kluyveromyces lactis, Laccaria bicolor, Lachancea thermotolerans, Latimeria chalumnae, Leishmania braziliensis, Leishmania donovani, Leishmania infantum, Leishmania major, Leishmania mexicana, Leptosphaeria maculans, Loa loa, Lodderomyces elongisporus, Loxodonta africana, Macaca fascicularis, Macaca mulatta, Magnaporthe oryzae, Malassezia globosa, Melampsora laricipopulina, Metarhizium acridum, Metarhizium robertsii, Meyerozyma guilliermondii, Micromonas sp., Mixia osmundae, Monodelphis domestica, Monosiga brevicollis, Mus musculus, Mycosphaerella graminicola, Myotis lucifugus, Naegleria gruberi, Naumovozyma castellii, Naumovozyma dairenensis, Nectria haematococca, Nematostella vectensis, Neosartorya fischeri, Neosartorya fumigata, Neospora caninum, Neurospora crassa, Neurospora tetrasperma, Nomascus leucogenys, Nosema ceranae, Oikopleura dioica, Oncorhynchus mykiss, Ornithorhynchus anatinus, Oryctolagus cuniculus, Oryza sativa subsp. indica, Oryza sativa subsp. japonica, Oryzias latipes, Ostreococcus lucimarinus, Otolemur garnettii, Pan troglodytes, Paracoccidioides brasiliensis, Paramecium tetraurelia, Pediculus humanus subsp. corporis, Penicillium chrysogenum, Penicillium marneffei, Perkinsus marinus, Phaeodactylum tricornutum, Phaeosphaeria nodorum, Physcomitrella patens subsp. patens, Phytophthora infestans, Phytophthora ramorum, Phytophthora sojae, Picea sitchensis, Pichia angusta, Pichia pastoris, Pichia sorbitophila, Piriformospora indica, Plasmodium falciparum, Plasmodium knowlesi, Plasmodium vivax, Plasmodium yoelii yoelii, Podospora anserina, Polysphondylium pallidum, Pongo abelii, Populus trichocarpa, Postia placenta, Pristionchus pacificus, Puccinia graminis f. sp. tritici, Pyrenophora teres f. teres, Pyrenophora tritici-repentis, Rattus norvegicus, Rhodotorula glutinis, Ricinus communis, Saccharomyces cerevisiae, Salmo salar, Salpingoeca sp., Sarcophilus harrisii, Scheffersomyces stipitis, Schistosoma mansoni, Schizophyllum commune, Schizosaccharomyces japonicus, Schizosaccharomyces pombe, Sclerotinia sclerotiorum, Serpula lacrymans var. lacrymans, Sordaria macrospora,

Sorghum bicolor, Spathaspora passalidarum, Sporisorium reilianum, Strongylocentrotus purpuratus, Sus scrofa, Taeniopygia guttata, Takifugu rubripes, Talaromyces stipitatus, Tetrahymena thermophila, Tetraodon nigroviridis, Tetrapisispora phaffii, Thalassiosira pseudonana, Theileria annulata, Theileria parva, Thielavia heterothallica, Thielavia terrestris, Torulaspora delbrueckii, Toxoplasma gondii, Tribolium castaneum, Trichinella spiralis, Trichomonas vaginalis, Trichophyton equinum, Trichophyton rubrum, Trichophyton tonsurans, Trichophyton verrucosum, Trichoplax adhaerens, Trypanosoma brucei brucei, Trypanosoma brucei gambiense, Trypanosoma congolense, Trypanosoma cruzi, Trypanosoma vivax, Tuber melanosporum, Uncinocarpus reesii, Ustilago maydis, Vanderwaltozyma polyspora, Verticillium albo-atrum, Verticillium dahliae, Vitis vinifera, Volvox carteri, Xenopus laevis, Xenopus tropicalis, Yarrowia lipolytica, Zea mays, and Zygosaccharomyces rouxii.

8 Spatial Sequence Correlation of FG and GF motifs

Within disordered regions of FG nups we found that the FG and GF motifs are colocalized. Excluding the motifs that were isolated from other motifs by greater than 100 AAs as noise, we found the distance to the nearest motif neighbor of a specific type from a given motif of a specific type. This measurement, averaged over all motifs in all the FG nups studied in this paper, resulted in the following average nearest neighbor separation values of 18.3 AAs between FG motifs, 24.0 AAs between GF motifs, and 21.7 AA between GF motifs and FG motifs.

References

[1] Murphy L, Wallqvist A, Levy R (2000) Simplified amino acid alphabets for protein fold recognition and implications for folding. Protein Engineering 13: 149–152.

[2] Yamada J, Phillips J, Patel S, Goldfien G, Calestagne-Morelli A, et al. (2010) A bimodal distribution of two distinct categories of intrinsically disordered structures with separate functions in fg nucleoporins. Molecular & Cellular Proteomics 9: 2205–2224.

[3] Bohm C, Railing K, Kriegel H, Kroger P (2004) Density connected clustering with local subspace preferences. In: Fourth IEEE International Conference on Data Mining. IEEE. pp. 27–34.
